# Proteogenomic Characterization Reveals Subtype‐Specific Therapeutic Potential for HER2‐Low Breast Cancer

**DOI:** 10.1002/advs.202513086

**Published:** 2025-12-27

**Authors:** Shouping Xu, Keda Yu, Lei Liu, Qin Wang, Xiaohui Wu, Yihai Chen, Guozheng Li, Xin Zhang, Bo Wei, Zitong Fu, Abiyasi Nanding, Zuxianglan Zhao, Lingbing Yang, Xingda Zhang, Jianyu Wang, Wantong Sun, Yi Hao, Zhongyi Cheng, Xiaojiang Cui, Hao Wu, Da Pang

**Affiliations:** ^1^ Department of Breast Surgery Harbin Medical University Cancer Hospital Harbin 150081 China; ^2^ Key Laboratory of Tumor Biotherapy of Heilongjiang Province Harbin Medical University Cancer Hospital Harbin 150081 China; ^3^ Department of Breast Surgery Shanghai Cancer Center and Cancer Institute Shanghai Medical College Fudan University Shanghai 200032 China; ^4^ Heilongjiang Clinical Research Center for Breast Cancer Harbin Medical University Cancer Hospital Harbin 150081 China; ^5^ Jingjie PTM Biolab (Hangzhou) Co. Ltd. Hangzhou 310018 China; ^6^ Department of Pathology Harbin Medical University Cancer Hospital Harbin 150081 China; ^7^ Department of Surgery Samuel Oschin Cancer Institute Cedars‐Sinai Medical Center Los Angeles CA 90048 USA

**Keywords:** breast cancer, HER2‐low, precision treatment, proteomics, tumor subtyping

## Abstract

The molecular heterogeneity and distinct features of HER2‐low breast cancer are poorly understood, limiting their precise management. To address this issue, longitudinal multiomic profiling of HER2‐low breast cancer is performed, including genomics, transcriptomics, proteomics, lactylomics, and phosphoproteomics, using 250 well‐characterized samples, and identified three proteomic subtypes: PS1 (estrogen response signaling enriched), PS2 (angiogenesis enriched), and PS3 (proliferation enriched and HER2‐high like). These three proteomic subtypes have distinct features and potential therapeutic strategies, namely, endocrine therapy, antiangiogenic therapy, and anti‐HER2 therapy, and are validated in external datasets and PDO models. In addition, a detailed description of the genomic characteristics and a map of the lactate modification landscape of HER2‐low breast cancer are provided. This research provides complementary information, reveals the molecular characteristics of HER2‐low breast cancer, and suggests potential precise therapeutic strategies for patients with this type of cancer.

## Graphical abstract

Multiomic profiling of HER2‐low breast cancer identifies three proteomic subtypes with distinct therapeutic strategies: endocrine, antiangiogenic, and anti‐HER2 therapies. Genomic and lactate modification landscapes are detailed, providing insights for precise management.

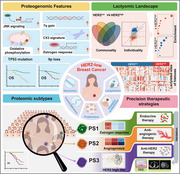

## 1 Introduction

Breast cancer is the most common cancer affecting women worldwide.^[^ ^1^ ^]^ In accordance with the widely used PAM50 typing criterion, breast cancers are categorized into different subtypes and treated on the basis of their hormone receptor (HR; estrogen receptor (ER) or progesterone receptor (PR)) status and human epidermal growth factor receptor 2 (HER2) status.^[^ ^2^ ^]^ HER2‐low breast cancer, characterized by IHC scores of 1+ or 2+/ISH‐, accounts for ≈50% of all breast cancer cases.^[^ ^3^, ^4^ ^]^ For HER2‐low breast cancer, due to the lack of precise therapeutic strategies, endocrine therapy and chemotherapy based on the PAM50 framework are still the cornerstone,^[^ ^5^, ^6^, ^7^ ^]^ but the overall benefits of ≥2‐line treatment are limited.^[^ ^8^ ^]^ In conclusion, the current therapeutic strategies are not precise enough to prolong HER2‐low breast cancer survival reliably, and the exploration of new precision treatment strategies is becoming increasingly urgent.

Recent clinical–molecular data reveal that HER2‐low or “ultra‐low” (IHC 0 with circumferential membrane staining), residual biologically active HER2 homo‐ and heterodimers that can continuously trigger downstream signaling of PI3K‐AKT‐mTOR and MAPK, maintaining tumor cell survival and proliferation. However, the signaling amplitude remains markedly below that observed in HER2‐high (IHC 3+ or 2+/ISH+) and is insufficient to drive efficient antibody–drug conjugate (ADC) internalization, explaining the limited efficacy of conventional HER2‐directed therapies.^[^ ^9^, ^10^, ^11^ ^]^ Recently, the DESTINY‐Breast04 trial confirmed for the first time that anti‐HER2 treatment (T‐Dxd, Next‐generation HER2 antibody‐drug conjugates) can significantly prolong the overall survival (OS; 22.9 vs 16.8, HR = 0.69) and progression‐free survival (PFS; 8.8 vs 4.2, HR = 0.36) of patients with HER2‐low breast cancer.^[^ ^12^ ^]^ The latest interim results of the DESTINY‐Breast06 trial not only confirm previous results (PFS: 13.2 vs 8.1, HR = 0.62) but also indicate that patients with HER2‐ultralow breast cancer can benefit from treatment with DESTINY‐Breast06 (PFS: 13.2 vs 8.3, HR = 0.78).^[^ ^13^ ^]^ The robust activity of T‐DXd in HER2‐low tumors is believed to be driven by three converging mechanisms:^[^ ^11^ ^]^ i) its cleavable tetrapeptide linker is exquisitely sensitive to the cysteine protease CTSL that is abundant in the acidic tumor microenvironment, enabling extracellular cleavage and release of membrane‐permeable DXd with potent bystander killing that bypasses limited ADC internalization; ii) compared with T‐DM1, T‐DXd possesses a higher drug‐to‐antibody ratio (DAR = 8) and employs the lipophilic payload DXd, markedly amplifying local cytotoxicity; iii) preclinical models demonstrate antitumor efficacy against HER2‐negative MDA‐MB‐468 xenografts, indicating that microenvironment‐dependent linker cleavage may be the dominant mechanism.

However, despite these encouraging results, the ORR of T‐Dxd in HER2‐low breast cancer patients is relatively low, with a value of only 40% in the DESTINY‐Breast04 trial.^[^ ^12^ ^]^ This situation may stem from the inadequate attention given to the intertumoral heterogeneity of HER2‐low breast cancer.^[^ ^14^ ^]^ In recent years, the indispensable role of multiomic technologies has been underscored;^[^ ^15^, ^16^, ^17^ ^]^ these technologies can not only be pivotal in revealing the mechanisms underlying cancer^[^ ^18^, ^19^, ^20^ ^]^ and dissecting internal heterogeneity^[^ ^21^, ^22^ ^]^ but also provide strong scientific support for precision medicine.^[^ ^23^, ^24^ ^]^ Therefore, exploring the heterogeneity of HER2‐low breast cancer via multiomics, identifying the biological characteristics of other HER2‐low breast cancer subgroups while screening out the subgroups sensitive to anti‐HER2 treatment, and further searching for potential therapeutic targets are particularly critical.

In this study, we provide a comprehensive multiomic dataset of HER2‐low breast cancer, including data collected by whole‐exome sequencing (WES), transcriptomics, proteomics, phosphoproteomics, and lactylomics. This study divided HER2‐low breast cancers into three subtypes (PS1, PS2, and PS3) and demonstrated the therapeutic potential of endocrine therapy, antiangiogenic therapy, and anti‐HER2 therapy, respectively, providing an important reference for devising precise treatment strategies for this group. Moreover, the project allowed a detailed description of the genomic characteristics and mapping of the lactylomic landscape of HER2‐low breast cancer, revealing new regulatory relationships in HER2‐low breast cancers. Overall, these findings not only reveal the potential driving mechanisms behind HER2‐low breast cancer but also facilitate a deeper and systematic understanding of the complexity associated with HER2‐low breast cancer from a multiomic perspective.

## 2 Results

### 2.1 Multiomic Profiling of HER2‐Low Breast Cancer

**FIGURE 1:**
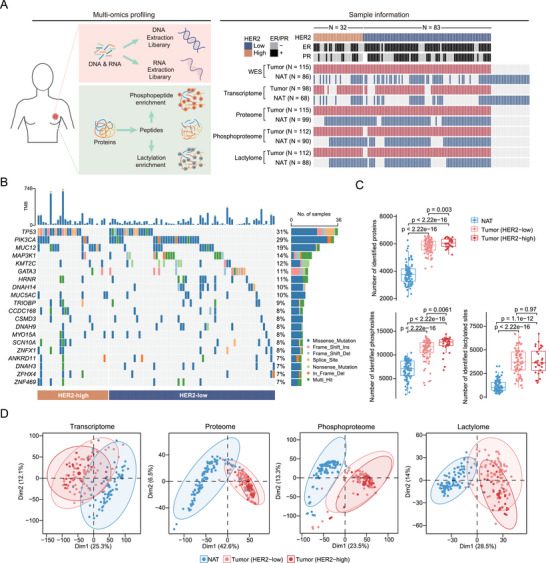
Multi‐omics landscape of HER2‐low breast cancer. A) Workflow and sample information for omics profiles. B) Genomic profile of the 115 breast cancer tumor samples with WES data. C) Boxplots showing the identified proteins, phosphosites, and lactylated sites in breast cancer tumor and normal adjacent tissues. D) Global transcriptome, proteome, phosphoproteome, and lactylome PCA plots of HER2‐high tumors, HER2‐low tumors, and NATs.

We collected 115 tumor and 135 normal adjacent tissue (NAT) samples from treatment‐naive Chinese breast cancer patients, including 83 HER2‐low tumor samples (IHC score 1+, *n* = 43; IHC score 2+ and ISH negative, *n* = 40) and 32 HER2‐high (IHC score 3+, *n* = 32) tumor samples. A schematic diagram of the experimental design is shown in **Figure** **1**A. Whole‐exome sequencing (WES) was conducted on 115 tumor and 86 NAT samples (Figure S1A, Supporting Information). RNA sequencing (RNA‐seq) was carried out on 98 tumor and 68 NAT samples (Figure S1B, Supporting Information). Proteome profiling was conducted on 115 tumor and 99 NAT samples (Figure 1C). For the posttranslational modification (PTM) profiles, phosphoproteomic and lactylomic approaches were used to analyze 112 tumor and 90 NAT samples and 113 tumor and 88 NAT samples, respectively (Figure 1C).

WES data from NATs were used as a reference to detect genetic variants in this cohort. The mean sequencing coverage in the hg38 reference genome was 204.48× for tumor tissues and 119.87× for NATs (Figure S1A, Supporting Information). Among the 115 tumor and 86 NAT samples, 10719 nonsilent mutations and 46109 silent mutations were detected. *TP53* (31%) and *PIK3CA* (29%) were the most frequently mutated genes in this cohort (Figure 1B).

For mass spectrometry (MS)‐based proteomic and PTM profiles, quality control (QC) with HeLa cell samples was conducted every one or two days. The median correlation coefficient among the QC samples was 0.98 for proteomics, 0.97 for phosphoproteomics, and 0.98 for lactylomics (Figure S1C, Supporting Information), which demonstrated the consistency and stability of the MS platform. Proteomic analysis of all the samples revealed a total of 8307 proteins, among which 6059 proteins were identified in more than half of the tumors or NATs (Table S2, Supporting Information). On average, we identified 5818 proteins in HER2‐low tumor tissues and 6061 proteins in HER2‐high tumors, which were significantly greater than the 3794 proteins identified in NATs (Figure 1C; Figure S1F, Supporting Information). Proteome quantification was conducted via the iBAQ algorithm, followed by normalization to the median of each sample (Table S2, Supporting Information). Among PTMs, a total of 43963 phosphosites and 18214 lactylation sites were identified (Figure 1C; Figure S1F, Supporting Information), corresponding to 7808 phosphoproteins and 1644 lactylated proteins. More phosphosites and lactylated sites were identified in both HER2‐high and HER2‐low tumors than in NATs (Figure 1C). We identified a greater number of phosphosites in HER2‐high tumors than in HER2‐low tumors, while the number of identified lactylated sites was similar between the two groups (Figure 1C). PTM data were quantified at the site level, followed by median normalization, which was consistent with the proteome data. Comparison with the proteomic profile showed that the subcellular locations of the phosphorylated proteins and lactylated proteins were similar (Figure S1E, Supporting Information). Additionally, a total of 2711 proteins were identified at the proteome, phosphoproteome, and lactylome levels (Figure S1F, Supporting Information).

RNA‐seq analysis revealed 12953 genes with fragments per kilobase of transcript per million fragments mapped (FPKM) values greater than 1, creating 5661 mRNA‒protein pairs for transcriptome‒proteome correlation analysis (Figure S1G,H, Supporting Information). The mRNA and protein levels showed moderate sample‐wise Pearson correlations, with medians of 0.54 and 0.46 for tumor tissues and NATs, respectively (Figure S1G, Supporting Information). Notably, tumors also presented greater genewise Pearson correlations than NATs did (Figure S1H, Supporting Information). Prerank gene set enrichment analysis (GSEA) revealed that genes with positive mRNA‒protein correlations were enriched in metabolism‐related and immune‐related pathways (Figure S1I, Supporting Information), which is consistent with previous reports.^[^ ^25^ ^]^ We then performed PTC (contribution ratios of PTMs and transcripts) analysis.^[^ ^26^ ^]^ Based on PTC values, proteins were divided into three categories: transcript dominant (TD) with PTC of 0–40%, balanced (BL) with PTC of 40–60%, and PTM dominant (PD) with PTC of 60–100% (Figure S1J, Supporting Information). For phosphorylation and lactylation, proteins in the PD category accounted for a higher proportion (39.7% and 48.6%) than the BL and PT categories (Figure S1K, Supporting Information). Ribosome and spliceosome are strongly enriched for lactylation‐dominant proteins, indicating that lactylation plays an important role in protein turnover regulation (Figure S1L, Supporting Information). Phosphorylation‐dominant proteins were related to cell adhesion, cytoskeleton regulation, and membrane trafficking, highlighting their central role in dynamic signaling and structural remodeling (Figure S1L, Supporting Information).

After appropriate sample QC and data preprocessing, we performed principal component analyses (PCAs) of the transcriptomic, proteomic, phosphoproteomic, and lactylomic data (Figure 1D). All datasets displayed distinct tumor and NAT separation, but there was considerable overlap between HER2‐high and HER2‐low tumors (Figure 1D). In summary, our study provides comprehensive data on breast cancer at the genomic, transcriptomic, proteomic, phosphoproteomic, and lactylomic levels to reveal the unique characteristics of HER2‐low breast cancer.

### 2.2 Proteogenomic Features of HER2‐Low Breast Cancer

Somatic (S)CNAs in this cohort were identified via GISTIC (Figure S2A, Supporting Information). The most common arm‐level deleted chromosomal regions in our cohort were 16q (53%), 8p (52%), 13p (51%), 22p (51%), 13q (45%), and 22q (45%), whereas the most common arm‐level amplified regions were 20q (65%), 19p (58%), 19q (58%), 16p (57%), 1q (56%), 20p (55%), 8q (48%), 14p (40%), 17q (38%), 22p (35%), and 22q (35%) (Figure S2B, Supporting Information). We further explored the impact of SCNA on mRNAs and proteins (Figure S2C, Supporting Information). For the cis effect, 1511 SCNA‐mRNA and 356 SCNA‐protein regulatory pairs were identified in this study, resulting in 221 SCNA‐mRNA‐protein cis regulatory pairs (Figure S2D, Supporting Information). Seven cancer‐associated genes (CAGs)^[^ ^27^ ^]^ with cis effects, including ERBB2 (17q12), CDK12 (17q12), IRF6 (1q32.2), DHX9 (1q25.3), PRCC (1q23.1), FH (1q43), and GNA13 (17q24.1), were identified. We further explored the protein‐level trans effects of the cis‐regulated genes and found that ERBB2 amplification had the strongest trans effects (Figure S2E, Supporting Information). This result further highlights the oncogenic driver role of ERBB2 amplification in breast cancer, which manifests as ERBB2 overexpression (HER2‐high). Notably, >90% of the HER2‐high samples showed ERBB2 amplification (log2 CN > 0.3) (Figure S2G, Supporting Information). On the other hand, these results indicate that HER2‐low breast cancer is more complex than HER2‐high breast cancer.

**FIGURE 2:**
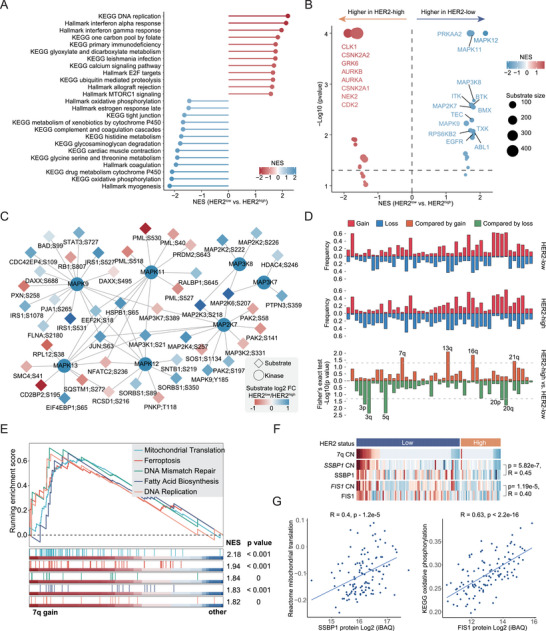
Proteogenomic features of HER2‐low breast cancer. A) GSEA of KEGG and hallmark pathways between HER2‐high and HER2‐low tumors on the basis of proteome data. B) GSEA of kinases between HER2‐high and HER2‐low tumors on the basis of phosphoproteome data. C) Kinase‐substrate network illustrating differences between HER2‐high and HER2‐low tumors in terms of phosphorylation abundance and kinase activity for MAPK signaling pathway members. D) Comparison of gene‐level CNAs between HER2‐low and HER2‐high tumors in this cohort. The upper plot illustrates the frequency of CNA events, and the lower plot illustrates the −log10 (*p* value) of each gene for the comparison of HER2‐low and HER2‐high tumors (two‐sided Fisher's exact test). E) GSEA plots showing the pathways enriched in tumors with 7q gain. F) Cis‐effects of 7q gain on SSBP1 and FIS1 in this study. G) Left: Scatter plot showing the correlation between SSBP1 protein abundance and mitochondrial translation score. Right: Scatter plot showing the correlation between FIS1 protein abundance and OXPHOS score. *p*‐values were derived from Spearman's correlation test.

We used proteomic and PTM data to explore the functional characteristics of HER2‐low breast cancer. Proteomic data from GSEA revealed enhanced expression of metabolic pathways such as oxidative phosphorylation, drug metabolism, cytochrome P450, and amino acid metabolism in HER2‐low breast cancer (**Figure** **2**A). Elevated levels of proliferation‐related pathways (DNA replication, E2F targets, and MTORC1 signaling) and immunity‐related pathways (interferon‐α response and interferon‐γ response) and decreased tight junction levels were observed in HER2‐high breast cancer (Figure 2A). The elevated proliferation in HER2‐high tumors was validated by the Ki67 IHC scores (Figure S2F, Supporting Information). Although the ESR1 level was not significantly different between HER2‐high and HER2‐low tumors, the estrogen response was enriched in HER2‐low tumors (Figure 2A; Figure S2G, Supporting Information). At the phosphoproteomic level, we found that kinases involved in the cell cycle (e.g., AURKA, AURKB, and CDK2) were activated in HER2‐high tumors, whereas kinases involved in JNK signaling (e.g., MAPK11, MAPK12, and MAP2K7) were activated in HER2‐low tumors (Figure 2B,C). Itah et al. reported that the development of HER2‐positive breast cancer was suppressed by the JNK signaling pathway,^[^ ^28^ ^]^ further highlighting that HER2 regulates the proteome and phosphoproteome in breast cancer. Interestingly, activation of the JNK signaling pathway was also reported to be associated with acquired resistance to HER2‐targeted therapy in breast cancer.^[^ ^29^ ^]^ These results indicate that HER2 and JNK are alternately activated in breast cancer and that the effects of the JNK pathway depend upon the status of HER2.

A comparison of the CNA landscape at the gene level between HER2‐high and HER2‐low tumors revealed that the frequencies of 3p, 3q, 20p, and 20q loss and 7q, 13q, 16q, and 21q gains were significantly different (Fisher's exact test, *p* < 0.05) (Figure 2D). Interestingly, we found that 7q gain was the only arm‐level SCNA event that more frequently occurred in the HER2‐low group (Figure 2D; Figure S2H, Supporting Information). GSEA revealed that these genes were associated with the upregulated pathways of mitochondrial translation, ferroptosis, DNA mismatch repair, and fatty acid biosynthesis (Figure 2E). To further investigate the effect of genes located at 7q, we prioritized genes that presented significant cis effects in this cohort. The copy numbers of *SSBP1* (7q34) and *FIS1* (7q22.1) were significantly correlated with their protein abundances (Figure 2F). Consistent with this, the protein levels of SSBP1 and FIS1 were increased in HER2‐low tumors (Figure S2I, Supporting Information). Moreover, we found that SSBP1 levels were positively correlated with mitochondrial translation and that FIS1 levels were positively correlated with oxidative phosphorylation (Figure 2G). In conclusion, these results indicate that the gain of genes located at 7q has potential oncogenic functions beyond ERBB2 amplification in breast cancer. In addition, we decomposed the CIN signatures of breast cancer.^[^ ^30^ ^]^ We found that HER2‐low tumors had a stronger CX3 signature but a lower CX2 signature than HER2‐high tumors did (Figure S2J, Supporting Information). CX3 is defined by an increase in CIN complexity compared to CX2 due to replication stress, impaired damage sensing, and impaired nucleotide excision repair (NER).^[^ ^30^ ^]^ Consistent with this, we observed downregulation of NER and other DNA repair pathways, except homologous recombination (HR), in HER2‐low breast cancer (Figure S2k, Supporting Information). In summary, our study provides a comprehensive proteomic characterization of HER2‐low tumors, including pathways, kinases, and genomic driver events.

### 2.3 Clinical Outcomes Associated with Genomic Alterations in HER2‐Low Breast Cancer

**FIGURE 3:**
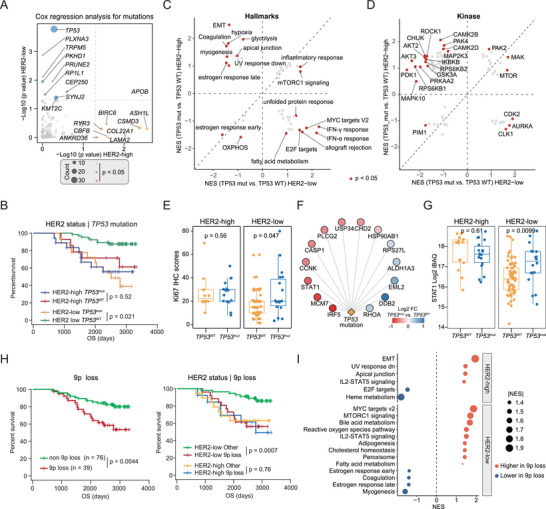
Prognosis associated with genomic alterations in HER2‐low breast cancers. A) Comparison of the effects of mutations on OS between HER2‐low and HER2‐high tumors. B) Kaplan–Meier curves of OS for patients with or without TP53 mutations with different HER2 statuses (two‐sided log‐rank test). C) 2D enrichment analysis revealed distinct hallmark pathway enrichment in different HER2 statuses. D) 2D enrichment analysis revealed distinct kinase enrichment in different HER2 statuses. E) Comparisons of Ki67 IHC scores between HER2‐high and HER2‐low tumors with or without TP53 mutation (Wilcoxon rank‐sum test). F) Associations between TP53 mutations and TP53 target gene products in HER2‐low breast cancer. G) Comparisons of STAT1 protein abundances between HER2‐high and HER2‐low tumors with or without TP53 mutation (Wilcoxon rank‐sum test). H) Kaplan–Meier curves of OS for patients with or without 9p loss with different HER2 statuses (two‐sided log‐rank test). I) GSEA results for the comparisons of tumors with and without 9p loss at the proteomic level.

ERBB2 amplification‐based ERBB2 overexpression was the dominant oncogenic event in HER2‐high breast cancer. We hypothesized that the impacts of genomic alterations on clinical outcomes might differ among breast cancer patients with distinct HER2 statuses. Cox regression analysis of mutations was performed in HER2‐high and HER‐low tumors (**Figure** **3**A). Interestingly, *TP53*, the most frequently mutated gene in this cohort, was associated with poor prognosis, which was observed only in HER2‐low breast cancer patients (Figure 3A,B). At the genomic level, *TP53*‐mutated tumors presented increased TMBs and CIN scores in both HER2‐high and HER2‐low tumors (Figure S3A,B, Supporting Information). Further proteomic analysis revealed that *TP53* mutations led to increased proliferation (E2F targets, MYC targets) and immune (IFN‐α/γ response) hallmarks in HER2‐low tumors but had the opposite effect in HER2‐high tumors (Figure 3C). At the phosphoproteomic level, the phosphorylation substrates of CDK2, AURKA, and CLK1 were enriched in *TP53* ^mut^ HER2‐low breast cancer (Figure 3D). Consistent with this, *TP53* mutation was associated with higher Ki67 IHC scores in HER2‐low tumors than in HER2‐high tumors (Figure 3E). We further analyzed the proteins significantly differentially expressed between *TP53*‐mutated tumors and tumors with wild‐type (WT) *TP53* in HER2‐low breast cancer patients, and 14 target gene products of *TP53* were identified (Figure 3F). Among them, STAT1, the core TF of the interferon response, was exclusively upregulated in TP53‐mutated tumors in HER2‐low breast cancer patients (Figure 3G). Additionally, the STAT1 protein expression levels were significantly correlated with patient survival (Figure S3C, Supporting Information). Similarly, the association between *KMT2C* mutation and poor survival was observed only in HER2‐low tumors (Figure 3A).

Cox regression analysis of arm‐level SCNA events revealed that 9p loss was associated with poor prognosis in patients with breast cancer (Figure S3D,H, Supporting Information). By further focusing on HER2‐low tumors, we found that 9p loss was also associated with poor prognosis (Figure S3E,H, Supporting Information). MTAP was the only gene located in 9p that presented a significant cis effect (Figure S3F, Supporting Information). HER2‐low breast cancer with 9p loss manifested as increased levels of proliferation hallmarks (MYC targets, MTORC1 signaling) and a decreased estrogen response (Figure 3I), similar to the impacts of *TP53* mutation. As expected, *TP53* mutation and 9p loss significantly cooccurred in this cohort (Figure S3F, Supporting Information). *CDKN2A/B*, located at 9p21.3, are important negative regulators of the cell cycle. Thus, aberrant proliferation resulting from loss of *CDKN2A/B* was the latent mechanism underlying the unfavorable prognosis for HER2‐low breast cancer patients, although no significant *cis/trans* regulation was observed. In addition, we found that the expression levels of SLC16A3 and EP300 were upregulated in HER2‐low tumors with 9p loss (Figure S3G, Supporting Information), suggesting the latent regulatory effect of 9p loss on lactylation in breast cancer.

### 2.4 Lactylomic Alterations in HER2‐Low Breast Cancer

**FIGURE 4:**
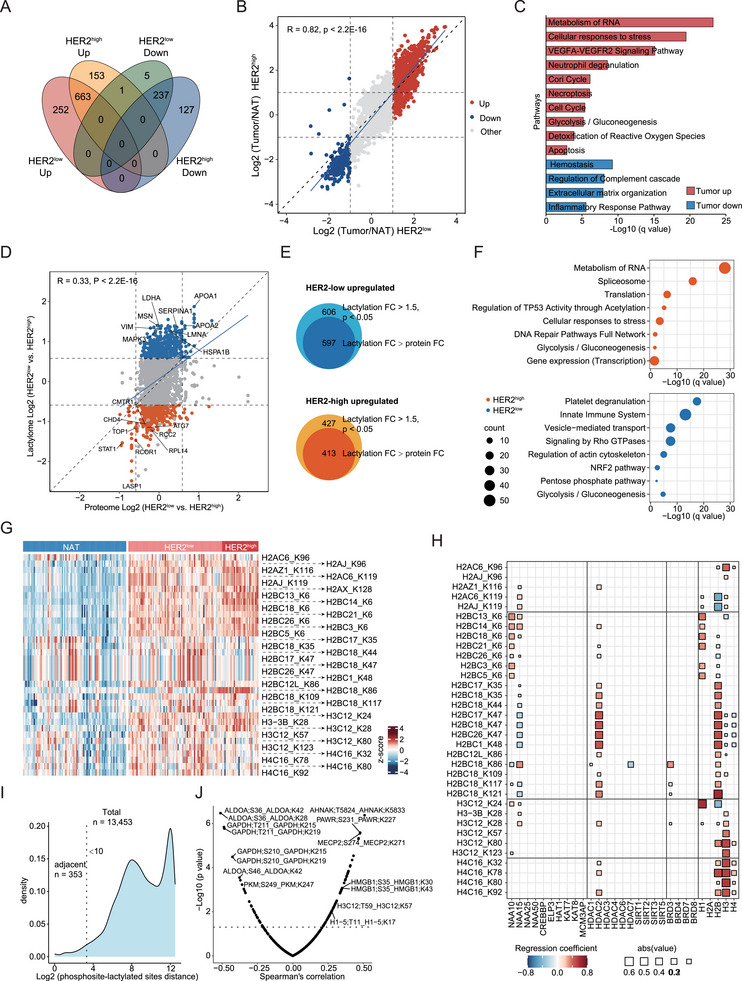
Lactylation alterations of HER2‐low breast cancers. A) Venn diagram showing the overlap of DEPs between HER2‐high tumors and HER2‐low tumors compared with NATs. B) Comparative analysis of protein alterations between HER2‐low tumors and NATs and between HER2‐high tumors and NATs. Spearman's correlation is shown. C) Pathway enrichment results of differentially expressed lactylation sites in tumors and NATs. D) Comparative analysis of the expression of global protein and lactylation levels between HER2‐low and HER2‐high tumors. E) Venn diagrams showing the overlap of HER2‐high and HER2‐low differentially expressed lactylation sites, and these sites are adjusted on the basis of the corresponding proteins. F) Bubble plots showing the pathways enriched by HER2‐low and HER2‐high differentially expressed proteins. G) Heatmap showing the core histone lactylation sites in tumors and NATs. H) Significant associations between core histone lactylation sites and acyltransferases, deacylases, and bromodomain‐containing proteins. I) The density curve of phosphosite‒lactylated site distances for all phosphorylation and lactylation‐modified proteins detected in this study. J) Correlation analysis of phosphosites and lactylated sites within 10 amino acids.

Lactylation has been reported to be a metabolic driver of tumors.^[^ ^31^ ^]^ However, its role in breast cancer is not clear. Here, LDHA (lactate producer), SLC16A1, and SLC16A3 (MCT1, MCT4, lactate transporters) were found to be significantly upregulated in both HER2‐high and HER2‐low breast cancer tumor tissues (Figure S4A, Supporting Information). Correspondingly, a greater number of lactylation sites were identified in the tumor tissues (Figure 1C). Breast cancer patients with higher SLC16A3 expression levels have a worse prognosis (Figure S4B, Supporting Information), indicating that lactylation plays a vital role in breast cancer. We identified 243 downregulated and 915 upregulated lactylated sites in HER2‐high breast tumors and 364 downregulated and 817 upregulated lactylated sites in HER2‐high breast tumors compared with NATs (Wilcoxon rank‐sum test, Benjamini–Hochberg adjusted *p* < 0.05, FC > 1.5) (**Figure** **4**A). HER2‐high and HER2‐low tumors showed high consistency when compared with NATs (Figure 4A,B). Thus, we compared all the breast cancer tumor tissues with NATs (Figure S4C, Supporting Information). The proteins with tumor‐upregulated lactylated sites were enriched in metabolism of RNA, cellular responses to stress, neutrophil degranulation, glycolysis/gluconeogenesis, the cell cycle, etc. The complement cascade, extracellular matrix organization, and inflammatory response pathways were enriched in the proteins related to tumor‐downregulated lactylated sites (Figure 4C).

Interestingly, we found apparent uncoupling between proteins and lactylation when comparing HER2‐low with HER2‐high tumors (Figure 4D). Almost all of the differentially lactylated sites between HER2‐high and HER2‐low tumors presented greater fold changes than did the corresponding proteins (Figure 4E). Overrepresentation analysis revealed that proteins with upregulated lactylation in HER2‐high tumors were enriched in the metabolism of RNA, spliceosome, translation, gene expression (transcription), glycolysis/gluconeogenesis, and DNA repair pathways (Figure 4F), while those in HER2‐low tumors were enriched in the innate immune system, signaling by Rho GTPases, regulation of the actin cytoskeleton, the NRF2 pathway, glycolysis/gluconeogenesis, and the pentose phosphate pathway (PPP) (Figure 4F). The differential abundances of enzymes involved in glucose metabolism are shown in Figure S4D (Supporting Information). These results indicate that lysine lactylation plays distinct regulatory roles in HER2‐high and HER‐low tumors.

The abundance of lactylation sites is determined mainly by lactylated protein substrates and enzymes (acyltransferases and deacylases). We further calculated the modification‒protein ratios (MPRs) in tumor and normal samples on the basis of the calculation method used for the protein‒transcript ratios (PTRs).^[^ ^32^, ^33^ ^]^ A total of 398 high‐MPR sites were identified in tumors and 395 in NATs, with 317 sites common to both (Figure S4E,F, Supporting Information). By focusing on the 98 tumor‐upregulated lactylation sites that presented high MPRs in both tumor tissues and NATs (Figure S4F, Supporting Information), overlactylation of glycolytic enzymes, including PFKL K677, PFKM K678, and PFKP K688, was observed (Figure S4G, Supporting Information). Most acyltransferases and deacylases were significantly altered in breast cancer samples (Figure S4H, Supporting Information), indicating that lactylation is widely regulated in breast cancer. Histone acetylation has been reported to be an epigenetic regulatory factor in recent years, but is aberrant in cancers.^[^ ^31^ ^]^ We detected more than 50 lactylated sites on histones H1, H2A, H2B, H3, and H4, among which most sites were significantly upregulated in the tumor tissues (Figure 4G; Figure S4I, Supporting Information). H2B K5 and H3 K27 lactylation was greater in HER2‐high tumors than in HER2‐low tumors (Figure 4G). Unlike histone acetylation,^[^ ^34^ ^]^ we found that histone lactylation showed positive correlation overall (Figure S4J, Supporting Information).

Lasso linear regression between histone acetylation sites and the protein abundances of acyltransferases, bromodomain‐containing proteins (BRDs), and deacylases was also performed (Figure 4H; Figure S5A, Supporting Information). This analysis revealed potential connections between NAA10, which is reported to display epsilon acetyltransferase activity,^[^ ^35^, ^36^ ^]^ and H2B K5 lactylation (H2BC3 K6, H2BC13 K6, H2BC14 K6, etc.) (Figure 4H). HDAC2 abundance was positively correlated with H2B lactylation, suggesting adaptive upregulation of HDAC2 to maintain the “lactylation–delactylation” balance of histone H2B^[^ ^37^ ^]^ (Figure 4H). We calculated Spearman's correlation coefficients of the core histone lactylation sites with hallmark pathway scores to explore the epigenetic regulation potential of histone lactylation (Figure S5B, Supporting Information). H2B lactylation at K34, K43, and K46 was positively correlated with TGFβ signaling and negatively correlated with NOTCH signaling and PI3K‐AKT‐MTOR signaling (Figure S5B, Supporting Information). H2B K5 and H3 K27 lactylation were positively correlated with proliferation and immune‐related pathways but negatively correlated with the estrogen response (Figure S5B, Supporting Information). In addition, H2B K5 lactylation (H2BC13 K6, H2BC14 K6) was upregulated in HER2‐low tumors with 9p loss (Figure S5C, Supporting Information). These results revealed the potential association between HER2 status‐related epigenetic regulation in breast cancer.

We then explored the lactylation‒phosphorylation crosstalk among phosphosites and lactylated sites after imputation and identified 13453 pairs, among which 353 adjacent pairs (<10 amino acids apart) were identified (Figure 4I). Notably, the glycolytic enzymes ALDOA and GAPDH and the PKM lactylation‐phosphorylation pairs were negatively correlated (Figure 4J). The histone adjacent modification pairs H1‐10 S31 | H1‐10 K23, H1‐4 T18 | H1‐4 K17, H1‐5 S18 | H1‐5 K17, and H1‐5 T11 | H1‐5 K17 were negatively correlated, but H3C12 T59 | H3C12 K57 and H3C12 T81 | H3C12 K80 were positively correlated (Figure 4J). H1‐5 T11 (H1.5 T10) has been reported to be maximally phosphorylated during the late G2 phase and M phase and to be dephosphorylated sharply thereafter,^[^ ^38^ ^]^ indicating that H1‐5 K17 lactylation might help drive progression through the G2M checkpoint. Four kinases (CREB1, MBD2, NFIC, and STAT1) and four other kinases (ACTR2, MAPK3, PAK2, and PRKDC) were identified to be comodified via lactylation and phosphorylation (nonadjacent pairs) (Figure S5D, Supporting Information). STAT1 K193 lactylation and T727 phosphorylation were highly positively correlated with STAT1 TF activity (Figure S5D, Supporting Information). PRKDC K2694 and K2908 lactylation and S893 and S4026 phosphorylation were highly correlated with PRKDC kinase activity (Figure S5D, Supporting Information).

### 2.5 Proteomic Intertumoral Heterogeneity Analysis of HER2‐Low Breast Cancer

The success of DESTINY‐Breast04 has attracted much attention in HER2‐low breast cancer research.^[^ ^39^ ^]^ HER2‐low breast cancer, as currently defined, should not be considered a distinct molecular subtype of breast cancer but rather a collection of heterogeneous tumors. A recent multi‐omic study of breast cancer was an important step in discriminating the molecular heterogeneity and distinct features of HER2‐low breast cancers.^[^ ^40^ ^]^ We applied non‐negative matrix factorization (NMF)‐based unsupervised clustering by integrating proteome, phosphoproteome, and lacylome data and divided the HER2‐low breast cancer into four clusters (Figure S6A–C, Supporting Information). These multi‐omic subtypes exhibited distinct biologic characteristics(Figure S6D, Supporting Information); however, the four multi‐omic subtypes did not differ in patient prognosis (Figure S6E, Supporting Information). Our aim was to derive molecular subtypes with genuine translational relevance, which are characterized by distinct biological features and strong clinical associations with patient prognosis. To this end, we further investigated whether proteome‐based subtyping alone could more effectively capture clinically meaningful heterogeneity.

**FIGURE 5:**
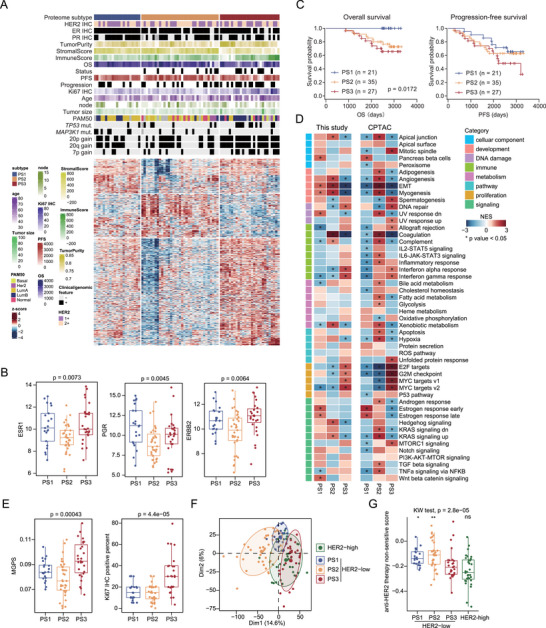
Proteomic subtypes of HER2‐low breast cancer. A) Relative abundances of upregulated proteins in the three proteomic subtypes and associations of proteomic subtypes with multiple variables. B) Comparisons of the protein abundances of ESR1, PGR, and ERBB2 among the three proteomic subtypes (Kruskal‒Wallis test). C) Kaplan–Meier curves of OS for the three proteomic subtypes (two‐sided log‐rank test). D) Heatmap representing hallmark pathway GSEA results for the three proteomic subtypes in this study and the CPTAC cohort. Asterisks indicate pathways with *p* < 0.05. E) Comparisons of MGPS and Ki67 IHC scores among the three proteomic subtypes (Kruskal‒Wallis test). F) PCA plot showing high similarity between the PS3 subtype and HER2‐high tumors on the basis of proteomic data. G) Comparisons of anti‐HER2 therapy insensitivity scores among three HER2‐low proteomic subtypes and HER2‐high tumors. The Kruskal‒Wallis test and the Wilcoxon rank‒sum test were performed. ^*^ *p* < 0.05, ^**^ *p* < 0.01, ns *p* ≥ 0.05.

The HER2‐low breast cancers were stratified into three proteomic subtypes (PSs): PS1 (*n* = 21), PS2 (*n* = 35), and PS3 (*n* = 27) via unsupervised clustering (Figure 5A; Figure S7A–C, Supporting Information). Although there was no significant difference in HER2/ER/PR IHC status among the three subtypes (**Figure** **5**A), the protein levels of HER2/ER/PR were significantly different among the three subtypes (Figure 5B). Notably, the proteomic subtypes significantly differed in OS (log‐rank test, *p* = 0.0172) but were similar in PFS (Figure 5C). GSEA was performed to identify the dominant pathways of each subtype (Figure 5D). Interestingly, we observed that estrogen response signaling was enriched in PS1 (Figure 5D), which was also validated at the transcriptome level (Figure S7D, Supporting Information). Moreover, transcriptomic data were used to infer the transcription factor activity of PGR, revealing the activation of PGR in PS1 (Figure S7E, Supporting Information). These findings indicate that the better prognosis of PS1 patients might be due to a better endocrine therapy response. To validate this hypothesis, we established a proteomic classifier to predict tamoxifen treatment outcome on the basis of proteomic data from Marchi et al.^[^ ^41^ ^]^ (Figure S7F, Supporting Information). The classifier divided our cohort into good and poor outcome groups (Figure S7G,H, Supporting Information). PS1 was predicted to be the most beneficial subtype from tamoxifen treatment (Figure S7G, Supporting Information). Notably, HER2 and ER IHC statuses were significantly associated with tamoxifen treatment outcome (Figure S7H, Supporting Information), which is consistent with previous reports,^[^ ^42^, ^43^ ^]^ verifying the high accuracy of this classifier model laterally.

An important feature of PS2 was high angiogenesis enrichment (Figure 5D). At the protein level, the levels of the vascular endothelial cell markers CD34 and CDH5 and the tumor vessel marker CD248 were significantly elevated in PS2 tumors (Figure S7I, Supporting Information). The composition of microvascular (mv) endothelial cells, derived from xCell analysis^[^ ^44^ ^]^ was also elevated in PS2 tumors (Figure S7J, Supporting Information), indicating the therapeutic potential of antiangiogenic agents in PS2 tumors. In addition, we observed a relatively high level of fibroblasts in PS2 (Figure S7J, Supporting Information), which was positively correlated with the mv endothelial cell level (Figure S7K, Supporting Information). Epithelial‒mesenchymal transition was also a feature of PS2 tumors (Figure 5D). The infiltration of fibroblasts can enhance the EMT of tumor cells in several kinds of tumors.^[^ ^45^, ^46^, ^47^ ^]^ The interplay of MV endothelial cells and fibroblasts^[^ ^48^ ^]^ might contribute to the EMT and angiogenesis features of PS2 tumors.

PS3 was enriched in proliferation‐related pathways (E2F targets, MYC targets, and the G2M checkpoint) and immune‐related pathways (interferon‐α response and interferon‐γ response) (Figure 5D), which was associated with the benefit from neoadjuvant therapy.^[^ ^49^ ^]^ In addition, interferon‐γ response was associated with immune checkpoint blockade treatment response.^[^ ^50^ ^]^ The proliferation feature of PS3 manifested as the highest Ki67 IHC score and the highest multigene proliferation score (MGPS) (Figure 5E). Interestingly, when dimensionality reduction was conducted via PCA on all the tumor proteome data, we observed a high degree of overlap between PS3 and HER2‐high breast cancer samples (Figure 5F). This result indicated that a cluster of HER‐low breast cancer was highly similar to HER2‐high breast cancer, indicating a potential benefit from anti‐HER2 therapy. Moreover, we extracted information on an anti‐HER2 therapy‐insensitive gene set from a gastric cancer proteomic study.^[^ ^51^ ^]^ Compared with PS1 and PS2 tumors, PS3 tumors had significantly lower anti‐HER2 therapy insensitivity scores, but the scores were higher than those of HER2‐high tumors (not significantly) (Figure 5G), suggesting the potential of using anti‐HER2 therapy for PS3 tumors.

Arm‐level CNA events varied among the three subtypes, reflecting profound genomic effects on the breast cancer proteome. Multiple arm‐level CNA events, including 20p gain, 20q gain, and 7p gain, were more frequently detected in PS3 (Fisher's exact test, *p* < 0.05; Figure 5A; Figure S8A, Supporting Information), accounting for 85.2%, 85.2%, and 48.1% of PS3 tumors, respectively. Among the three proteomic subtypes, PS3 presented the highest CIN and Sig4 (APOBEC‐related signature) activities (Figure S8B,C, Supporting Information). These results further highlight the similarity of PS3 HER2‐low and HER2‐high tumors.

To improve the clinical availability of our proteomic subtypes, we established a support vector machine (SVM) model based on a 10‐feature panel (Figure S8D, Supporting Information). High accuracies, with areas under the receiver operating characteristic curve (AUROC) values of 0.97 and 1.00, were achieved in the training set and test set, respectively (Figure S8E,F, Supporting Information). We applied this model to the HER2‐low breast cancer proteomic data in the CPTAC cohort,^[^ ^52^ ^]^ which yielded similar hallmark features to those observed in our cohort (Figure 5D).

### 2.6 PTM Heterogeneity in HER2‐Low Breast Cancer

**FIGURE 6:**
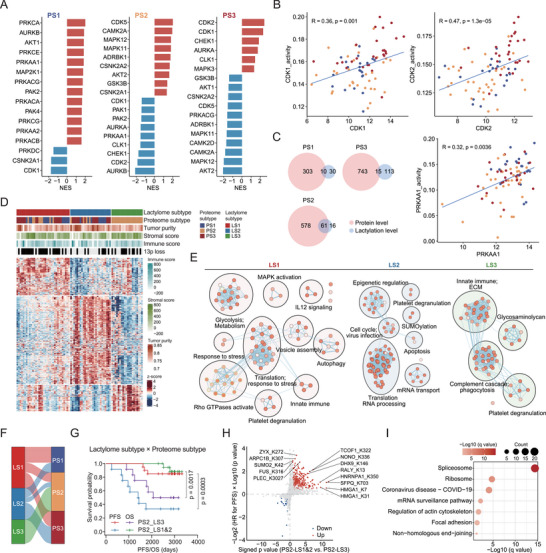
Deciphering the PTM heterogeneity in HER2‐low breast cancer. A) Evaluation of kinase activities by phosphoproteome‐based GSEA in tumors across the three proteomic subtypes. B) Scatter plots showing the correlations between protein abundances and kinase activities of CDK1, CDK2, and PRKAA1 (Spearman's correlation). C) Venn diagrams showing the overlap of PS‐specific upregulated lactylation sites and these sites adjusted by their corresponding proteins. D) Relative abundances of upregulated lactylation sites in the three lactylation subtypes and associations of lactylation subtypes with multiple variables. E) Network representation of pathways significantly enriched in lactylated sites across lactylation subtypes. Pathways are indicated as nodes, and edges indicate shared genes. Network clusters were summarized to reduce pathway redundancies, and the common functional themes were manually annotated as text. F) Sankey diagram depicting the comparison of proteomic subtypes and lactylation subtypes. G) Kaplan–Meier curves of OS and PFS for the three lactylation subtypes in PS2 tumors. H) Scatter plot showing the global differentially lactylated sites between PS2‐LS1&2 and PS2‐LS3 and Cox regression analysis of PFS. The x‐axis shows the log2 FC multiplied by ‐log10(*p*‐value). The y‐axis shows log2 HR multiplied by ‐log10 (*p*‐value). I) Enrichment analysis revealed that lactylation at 250 sites was downregulated in PS2‐LS3 compared with PS2‐LS1&2 and associated with an unfavorable prognosis.

We further explored the PTM heterogeneity in HER2‐low breast cancers. Comparison of the phosphoproteome among the three proteomic subtypes revealed subtype‐specific activated kinases, including PKA (PRKAA1, PRKAA2, PRKACB), PKC (PRKCA, PRKCE), AKT1, AMPK (PRKACA, PRKACG), and PAK (PAK2, PAK4) for PS1; CAMK2A, CK2A1 (CSNK2A1, CSNK2A2), and GSK3B for PS2; and CDK1, CDK2, CHEK1, and CLK1 for PS3 (**Figure** **6**A). Among these kinases, CDK1, CDK2, and PRKAA1 showed a significantly positive correlation between protein abundances and kinase activities (Figure 6B). With regard to lactylation, we identified 86, 312, and 192 subtype‐specific upregulated lactylation sites in PS1, PS2, and PS3, corresponding to 40, 77, and 128 proteins, respectively (Figure 6C). Interestingly, we found that the alteration of lactylation was consistent with the alteration of cognate proteins in PS2, but this alteration was uncoupled in PS1 and PS3 (Figure 6C). As a result, we further explored the heterogeneity of lactylation in HER2‐low breast cancer by consensus clustering of lactylated site abundances, which revealed three stable lactylomic clusters (Figure 6D; Figure S9A–C, Supporting Information). Unlike the proteomic subtypes, the lactylomic subtypes significantly differed in PFS (log‐rank test, *p* = 0.00172) but were similar in OS (Figure S9D,E, Supporting Information). However, we discovered that genomic alterations exhibited high variability within each lactylomic subtype, resulting in few distinct genomic alteration features for each subtype (Figure S9F, Supporting Information). Proteins involved in pathways such as glycolysis, MAPK activation, response to stress, Rho GTPase activation, and autophagy were strongly modified in LS1. The cell cycle, epigenetic regulation, translation, and apoptosis‐related protein lactylation were upregulated in LS2. LS3 was characterized by the complement cascade, the ECM, and immune signaling protein lactylation (Figure 6E).

Half of the PS1, PS2, and PS3 tumors were classified as LS1, LS3, and LS2, respectively (Figure 6F). Interestingly, we found that the lactylomic data further divided PS2 tumors into two groups with different prognoses. Compared with PS2‐LS1&2, PS2‐LS3 resulted in longer survival (Figure 6G). These results indicate that lactylomic analysis is an important complementary analysis in breast cancer research. Among the 540 lactylation sites downregulated (Wilcoxon rank‐sum test, *p* < 0.05, FC > 1.5) in PS2‐LS3 compared with PS2‐LS1&2, 250 sites were associated with an unfavorable prognosis (HR[PFS] > 1, *p* < 0.05) (Figure 6H). Proteins corresponding to these sites were enriched in the spliceosome, ribosome, regulation of actin cytoskeleton, focal adhesion, and nonhomologous end‐joining pathways (Figure 6I), highlighting the impacts of lactylation on tumor malignancy through biological processes beyond metabolism. Phosphoproteome‐based GSEA revealed that AURKB was inactivated in LS3 (Figure S9G,H, Supporting Information) and in PS2. PS2‐LS3 had lower AURKB kinase activity than PS2‐LS1&2 did (Figure S9I, Supporting Information). These results indicated that AURKB kinase activity, which is associated with patient prognosis (Figure S9J, Supporting Information), is regulated by lactylation. In addition, we further explored the latent lactylation targets in the LS2 upregulated sites, as described in Figure S9K (Supporting Information). A total of 35 lactylation sites, corresponding to 32 proteins, were identified. Among these, H2BC14 K6, H2BC14 K6, and H3C13 K28 were associated with proliferation and the immune response (Figure S5B, Supporting Information). PRKDC K2694 and K2908 were significantly correlated with PRKDC kinase activity (Figure S5D, Supporting Information). In addition, MAPRE1 and STMN1 were associated with microtubules, and their glycosylation might be related to paclitaxel resistance.

### 2.7 Clinical Verification of PS Subtyping based on Immunohistochemistry

**FIGURE 7:**
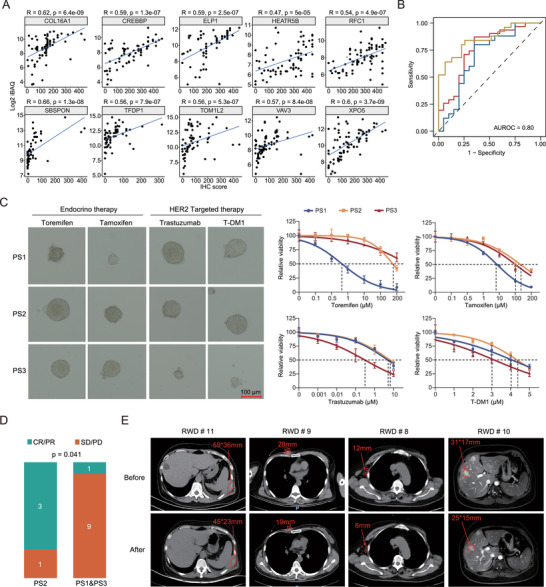
Exploration of clinical application of HER2‐low breast cancer subtypes. A) Scatter plots showing the correlations between the IHC scores and proteomic abundances of the 10 proteomic subtype features. B) ROC curves for proteomic subtype assignment on the basis of IHC scores. C) The therapeutic effects of endocrine therapy (toremifene/tamoxifen: 0–200 µm) and anti‐HER2 therapy (trastuzumab/T‐DM1: 0–100 µg mL^−1^) in the predicted PS1 (*n* = 3), PS2 (*n* = 3) and PS3 (*n* = 3) PDO models (left: representative bright‐field microscopy images; right: dose‒response curves). D) Comparison of antiangiogenic therapy outcomes between the predicted PS2 and PS1&PS3 patients on the basis of real‐world data (RWD). E) Imaging information for the antiangiogenic therapeutic effect for PS2 patients is shown in Figure 7D.

To further verify the clinical accessibility of our HER2‐low subtype, we collected 110 samples, including 76 samples mentioned previously for IHC assessment, along with an additional 20 patient‐derived organoid (PDO) model samples and 14 samples from real‐world retrospective data (RWD). IHC assessment was performed on 10 protein features used for proteomic subtyping assignment via the SVM model. The IHC scores and proteomic abundances showed median correlations among the 10 protein features, ranging from 0.47 to 0.66 (**Figure** **7**A). We applied the SVM model to predict the proteomic subtypes of the 110 samples via the use of IHC scores (Table S8, Supporting Information). For the 76 samples with proteomic profiles, the IHC score‐based proteomic subtyping yielded an AUROC of 0.80 (Figure 7B). Moreover, to verify the therapeutic potential of HER2‐low breast cancer proteomic subtyping, PDO models for each proteomic subtype were applied to conduct drug susceptibility assays (Figure 7C). PS1, PS2, and PS3 were more susceptible to toremifen and tamoxifen (endocrine therapy), bevacizumab and apatinib (antiangiogenic therapy), and trastuzumab and T‐DM1 (anti‐HER2 therapy), respectively (Figure 7C). These results confirmed the therapeutic potential of the HER2‐low proteomic subtypes. Moreover, the 14 patients who received anti‐angiogenic therapy (apatinib, anlotinib, bevacizumab) in our cohort were classified into the PS1 subtype (*n* = 6), PS2 subtype (*n* = 4), and PS3 subtype (*n* = 4). Compared with the PS1 and PS3 patients, PS2 patients had better outcomes (Figure 7D,E). In the PS2 subtype, 3 patients out of 4 achieved complete remission (CR) or partial remission (PR) on the basis of imaging information for the metastatic lesion according to WHO^[^ ^53^ ^]^ or RECIST criteria (version 1.1)^[^ ^54^ ^]^ (costal metastasis PR for RWD #11, chest wall metastasis PR for RWD #9, axillary lymph node metastasis PR for RWD #8, and liver metastasis SD for RWD #10), whereas in the PS1 and PS3 subtypes, only 1 patient out of 10 achieved CR or PR. These results further support the antiangiogenic therapeutic potential of PS2 HER2‐low tumors.

## 3 Discussion

Recently, the concept of HER2‐low cancer has been proposed, accounting for over half of all breast cancer patients.^[^ ^55^, ^56^ ^]^ Within the classical PAM50 taxonomy, HER2‐low breast cancers occupy a marginalized niche: their intrinsic pathogenesis and actionable targets remain obscure, and therapeutic decisions are dictated largely by HR status, defaulting to endocrine therapy and chemotherapy—an approach that is fundamentally imprecise. Here, we comprehensively revealed the molecular nature and interpatient heterogeneity among HER2‐low breast cancers on the basis of multiomic analyses (genomics, transcriptomics, proteomics, lactylomics, phosphoproteomics) and clinicopathological features. The activation of JNK signaling in HER2‐low breast cancer suggests it acts as a compensatory survival pathway, supporting the rationale for combining JNK inhibitors with HER2‐targeted therapy. Furthermore, we successfully divided all HER2‐low populations into three potential subtypes (PS1/2/3) and proposed potential precise treatments for them (PS1: endocrine therapy; PS2: antiangiogenic therapy; PS3: anti‐HER2 therapy). The therapeutic effects were confirmed in a subsequent PDO model and with retrospective analysis of real‐world data. In addition, we provided a detailed description of the genomic characteristics and mapped the lactate modification landscape of HER2‐low breast cancer. Our research provides additional insights into the mechanisms of breast cancer tumorigenesis and supports the development of precise PS‐based treatment strategies for HER2‐low breast cancer.

The first HER2‐targeted treatment to be extensively studied for HER2‐low disease was trastuzumab. NSABP B‐47 was a phase 3 trial in which 3270 HER2‐low breast cancer patients were subjected to chemotherapy with or without trastuzumab in a randomized manner.^[^ ^57^ ^]^ Unfortunately, there was no difference in invasive disease‐free survival (iDFS) between the groups, irrespective of hormone receptor status, HER2 IHC expression, or lymph node involvement, at a median follow‐up of 46 months.^[^ ^57^ ^]^ Similarly, studies have shown little benefit with pertuzumab^[^ ^58^ ^]^ and trastuzumab‐emtansine (T‐DM1)^[^ ^59^ ^]^ for the treatment of patients with HER2‐low breast cancer. On the basis of these findings, the use of HER2‐targeted agents is limited to patients with HER2‐positive tumors. With the publication of the results of the DESTINY‐Breast04 trial,^[^ ^60^ ^]^ the United States Food and Drug Administration (US FDA) approved T‐DXd in August 2022 for HER2‐low advanced breast cancer patients who have received ≥ 1 chemotherapeutic agent, regardless of hormone receptor status, and the use of HER2‐targeted agents for HER2‐low breast cancer re‐emerged and gained momentum. Furthermore, studies such as DESTINY‐Breast 06 are evaluating whether T‐DXd can be used in the first‐line setting for HR+/HER2‐low advanced breast cancer resistant to endocrine therapy.^[^ ^13^ ^]^ However, research on HER2‐targeted treatment in HER2‐low early breast cancer patients is still lacking. Our study focused on HER2‐low early breast cancer patients, identified the PS subtype, and revealed the anti‐HER2 therapeutic potential of PS3, which was verified in the PDO model. In addition, in our study, the proportion of PS3 subtypes with anti‐HER2 therapeutic potential was 32.53%, which is consistent with the 40% objective response rate of T‐DXd in the DESTINY‐Breast04 trial.^[^ ^60^ ^]^ Our research provides a theoretical basis for the application of HER2‐targeted treatment in HER2‐low early breast cancer.

To date, most studies have characterized HER2‐low breast cancer utilizing clinicopathological and prognostic information,^[^ ^55^, ^61^, ^62^ ^]^ and a large comprehensive multiomic dataset of HER2‐low breast cancers is still lacking. Although some studies have attempted to elucidate molecular characteristics, compared with our dataset, they all have limitations. Studies derived from the TCGA dataset may not be suitable for current studies of HER2‐low breast cancers because of possible interference with outdated criteria on the basis of the IHC HER2 subtype.^[^ ^63^ ^]^ Our team has divided HR‐/HER2‐low breast cancer patients into three subtypes on the basis of transcriptomic data and proposed potential therapeutic targets for each subtype.^[^ ^64^ ^]^ Similarly, our collaboration team members have also classified HR+/HER2‐negative patients into four subtypes on the basis of multiomic data.^[^ ^65^ ^]^ However, as a potential independent group, the HR status in HER2‐low breast cancer is an important part of its complex heterogeneity, which cannot be avoided. Segregating HER2‐low cancers on the basis of HR status may not provide a comprehensive and systematic understanding of the heterogeneity of HER2‐low cancers. In addition, although potential therapeutic targets with subtype specificity have been proposed at the theoretical level, there is a lack of experimental validation to support their reliability. Dai et al. included HER2‐low breast cancer patients regardless of HR status in their sudy,^[^ ^40^ ^]^ but the proposed subdivision (basal‐like and nonbasal‐like) was essentially similar to that of TNBC patients and HR+HER2‐low patients, and the potential targets were not thoroughly explored and verified; thus, guidance for HER2‐low treatment was limited. Our study included both HR+ and HR‐ HER2‐low breast cancer patients, regardless of HR status, on the basis of multiomic features, and, for the first time, included lactomics and photogenomics to comprehensively and systematically elucidate their internal heterogeneity. We established proteomic subtypes and clarified their biological characteristics on the basis of multiomic data. Potential subtype‐specific therapeutic targets were proposed, which were validated by the PDO model and retrospective analysis of real‐world data from patients in our cohort. Notably, our research provides valuable data that could expand our understanding of HER2‐low breast cancer biology.

Knowledge of the specific genomic characteristics of HER2‐low breast cancer is still lacking. Previous studies have shown that HER2‐low tumors have a greater copy number of ERBB2 than HER2‐0 tumors do, but there are no significant differences in other gene mutations, CNVs, or TMB.^[^ ^61^ ^]^ Our research indicates that the impacts of genetic variations are influenced by the status of HER2.^[^ ^66^ ^]^ On the one hand, HER2‐low tumors presented increased NER‐impairment‐related CX3 signatures and, more frequently, 7q gain, which impacted mitochondrial function in breast cancer. On the other hand, the association between *TP53* mutations and poor prognosis is more pronounced in HER2‐low tumors than in HER2‐high tumors, which is consistent with previous observations.^[^ ^67^ ^]^ Mechanistically, *TP53* mutations in HER2‐low breast cancer lead to enhanced proliferation and an altered immune response, which are not observed in HER2‐high tumors. These findings might be due to the inherently strong proliferative capacity and immune response of HER2‐high tumors. The observations related to the loss of 9p are similar to those related to *TP53*. Taken together, our results provide a comprehensive comparison of HER2‐low and HER2‐high breast cancer tumors at the genomic level, revealing some HER2‐dependent genomic variation, which helps improve our understanding of HER2‐low breast cancer.

Lactate has been reported to be a metabolic driver of tumors.^[^ ^68^ ^]^ Zijian et al. presented the lactylation landscape and revealed the extensive regulatory effect of lactylation on cellular metabolism in hepatocellular carcinoma.^[^ ^37^ ^]^ Recent studies highlighted the nonmetabolic regulatory functions of lactylation, such as in DNA repair^[^ ^69^ ^]^ and transcriptional regulation.^[^ ^70^ ^]^ Our study provides the first large‐scale lactylomic profile of both histone and nonhistone proteins in patients with breast cancer. Integrated analysis of lactylomic data with other omic data revealed a potential regulatory effect on gene expression, kinase activity, and cellular metabolism. To explore the potential regulatory role of lactoacylation in HER2‐low breast cancers, we performed unsupervised clustering of lactylated site abundances globally, revealing three stable lactylomic proteome clusters (LS1/2/3). There were significant differences in PFS among the three clusters, each of which had unique biological characteristics. Recent studies have revealed that many DNA damage response proteins involved in therapy resistance — such as NBS1,^[^ ^69^ ^]^ MRE11,^[^ ^71^ ^]^ XRCC1,^[^ ^72^ ^]^ and XLF^[^ ^73^ ^]^ — undergo lysine lactylation. In our cohort, lactylation of PRKDC was significantly associated with a shorter PFS, consistent with the trend observed the recent reports. These findings suggest that lactylation constitutes a key regulatory layer in DNA repair pathways that may promote treatment resistance. Therapeutic strategies aimed at inhibiting lactylation or directly targeting PRKDC could therefore help overcome resistance and prolong clinical benefit.

However, this study has certain limitations. When investigating the molecular characteristics of the HER2‐low breast cancer, the present study included only HER2‐high for comparison and lacked an HER2‐negative cohort. Consequently, important features that distinguish HER2‐low from HER2‐negative disease may have been missed. In future work, we will assemble a larger, inclusive cohort that spans the full HER2 continuum—negative, low, and high—to fully clarify the continuous spectrum of HER2 expression and to identify alterations that are truly specific to HER2‐low breast cancer; this will be a central focus of our subsequent studies. Additionally, the present findings were derived exclusively from a Chinese patient cohort. Consequently, the generalizability of these results to populations of different genomic backgrounds or ethnic ancestries may be constrained by potential inter‐population genetic and environmental heterogeneity.^[^ ^74^ ^]^

In conclusion, based on a large‐scale multiomic analysis, our study has revealed the heterogeneity of HER2‐low breast cancer from the perspective of its molecular features. We further propose subtype‐specific precision treatment strategies that may pave the way for improved application of precision medicine in HER2‐low breast cancer.

## 4 Experimental Section

### Clinical Specimens

This study included a total of 115 breast cancer samples and 135 NAT samples for multiomics, 14 samples from RWD, and 17 extra breast cancer tumor samples for constructing PDO models, which were prospectively collected from Harbin Medical University Cancer Hospital (Harbin, China). This study was approved by and conformed to the clinical research guidelines of the Research Ethics Committee of Harbin Medical University Cancer Hospital (Protocol number: KY2023‐84). Written informed consent was obtained from all patients. The clinicopathological features assessed in this study included age, tumor size, tumor histologic type, histologic grade, lymph node status, and ER, PR, HER2, and Ki67 statuses (Table S1, Supporting Information), for which reagents, antibody clones, and cut‐offs followed the 2023 ASCO/CAP guidelines.^[^ ^75^ ^]^ OS was defined as the interval between surgery and death.

### DNA and RNA Coextraction and Sequencing

WES libraries were prepared via SureSelect Human All Exon v6 (Agilent) with probes capturing the whole exome and sequenced on a DNB‐seq platform (BGI Genomics, China). Genomic alterations, including single‐base substitutions (single‐nucleotide variants), short and long insertions/deletions (indels), and copy number alterations, were assessed. The reads with the adaptor were removed from the raw rdata before alignment using SOAPnuke (v2.2.1). Clean data were mapped to the human reference genome (hg38) using Burrows‐Wheeler Aligner (BWA v 0.7.17) BWA‐MEM algorithm. Alignment files were sorted and converted to BAM format with SAMtools (v1.3.1), and PCR duplicates were marked using Picard/GATK MarkDuplicates (v4.4.0). Somatic single‐nucleotide variations and small insertions and deletions (InDels) were detected using MuTect2 (v4.4.0) and were annotated using Funcotator (v4.4.0).

RNA‐seq was also performed on a DNB‐seq platform, and 150 bp paired‐end reads were generated. The reads with the adaptor or poly‐A sequences were removed from the raw data before alignment using SOAPnuke (v2.2.1). The clean reads were aligned to the reference genome (GRCh38.p13 assembly) via Bowtie2 (ver 2.3.4.3).^[^ ^76^ ^]^ The average mapping ratio to the genome across samples was 97.94%, and the average mapping ratio with gene regions was 75.04%. The gene expression levels were quantified by RSEM (ver 1.3.1). The relative expression levels of the transcripts or genes were measured via a normalized metric, namely, FPKM.

### Protein Extraction

The frozen tissue samples were ground with liquid nitrogen into powder after being washed with PBS. Four volumes of 10% TCA/acetone were added, and the samples were incubated at −20 °C for 5 h. After centrifugation at 6500 × g for 5 min, the precipitate was washed three times with precooled acetone. Lysis buffer (1% SDS, 1% protease inhibitor cocktail, 1% phosphatase inhibitor, 3 µm TSA, 50 mm NAM) was added to the precipitate, followed by sonication three times on ice via a high‐intensity ultrasonic processor (Scientz). The remaining debris was removed by centrifugation at 12000 × g and 4 °C for 10 min. Finally, the supernatant was collected, and the protein concentration was determined with a BCA kit according to the manufacturer's instructions.

### Trypsin Digestion

The protein solutions were adjusted to the same volume by adding lysis buffer. An equal volume of precooled acetone was added, the mixture was vortexed, and four volumes of precooled acetone were added. After centrifugation at 4500 × g for 5 min, the precipitate was washed twice with precooled acetone. The dried precipitate was dissolved in 200 mm TEAB and digested with trypsin (1:50 trypsin‐to‐protein ratio) overnight. The tryptic peptides were reduced with 5 mM dithiothreitol for 30 min at 56 °C and alkylated with 11 mm iodoacetamide for 15 min at room temperature in the dark. Finally, the peptides were desalted on a C18 SPE column and lyophilized for affinity enrichment or LC‒MS/MS analysis.

### Phosphopeptide Affinity Enrichment

Trypsinized peptide samples were initially mixed with IMAC microsphere suspensions in loading buffer (50% acetonitrile/0.5% acetic acid) with shaking. To eliminate nonspecifically bound peptides, the microspheres underwent sequential washes with 50% acetonitrile/0.5% acetic acid and 30% acetonitrile/0.1% trifluoroacetic acid. For phosphopeptide elution, a 10% NH4OH elution buffer was added, and the phosphopeptides were released through vibration. The phosphopeptide–containing supernatant was collected and dried via lyophilization prior to LC‒MS/MS analysis.

### Affinity Enrichment for Lysine Lactylation

Tryptic peptides were redissolved in 200 µL of enrichment buffer (100 mm NaCl, 1 mm EDTA, 50 mm Tris‐HCl, 0.5% NP‐40; pH 8.0) and incubated with prewashed antibody beads (PTM‐1404, PTM Bio) at 4 °C overnight with gentle shaking. The beads were then washed with enrichment buffer four times and deionized water two times to remove the nonspecifically adsorbed peptides. Lactylated peptides were eluted with 0.1% TFA three times. The eluted lactylated peptides were collected and then desalted via C18 Zip Tips according to the manufacturer's instructions. After desalting, the lactylated peptides were lyophilized for further LC‒MS/MS analysis.

### LC‒MS/MS for Global Proteomic Analysis

The trypsin‐digested peptides were suspended in solvent A (0.1% formic acid, 2% acetonitrile in water) and then directly injected into a self‐made reverse‐phase analytical column (25 cm × 100 µm i.d.). On a nanoElute UHPLC system (Bruker Daltonics), peptide separation was achieved with a gradient elution: solvent B (0.1% formic acid in acetonitrile) increased from 6% to 24% over 70 min, from 24% to 35% in 14 min, then to 80% in 3 min, and remained at 80% for the final 3 min, all at a steady flow rate of 450 nL/min. Subsequently, the peptides were introduced into a timsTOF Pro (Bruker Daltonics) mass spectrometer via a capillary source. The applied electrospray voltage was 1.75 kV. The TOF detector was used to analyze precursors and fragments, with MS/MS scans covering 100–1700 m/z. The timsTOF Pro operated in PASEF mode, selecting precursors with charge states of 0–5 for fragmentation, and acquiring 10 PASEF‐MS/MS scans per cycle. Dynamic exclusion was set at 30 s.

### LC‒MS/MS for Phosphoproteomic Analysis

The enriched tryptic peptides were dissolved in solvent A and directly loaded onto a custom‐made reversed‐phase analytical column (25 cm length, 100 µm i.d.). The mobile phase consisted of solvent A (0.1% formic acid, 2% acetonitrile/in water) and solvent B (0.1% formic acid in acetonitrile). Peptides were separated with the following gradient: 0–76 min, 2–22% solvent B; 76–82 min, 22–35% solvent B; 82–86 min, 35–90% solvent B; 86–90 min, 90% B; at the entire procedure was performed a constant flow rate of 450 nL min^−1^ on a NanoElute UHPLC system (Bruker Daltonics). The peptides were subjected to ionization with a capillary source followed by mass spectrometry on a timsTOF Pro mass spectrometer. The electrospray voltage applied was 1.7 kV. The precursors and fragments were analyzed at the TOF detector, with an MS/MS scan range from 100 to 1700. The timsTOF Pro was operated in parallel accumulation serial fragmentation (PRM‐PASEF) mode. Precursors with charge states of 0–5 were selected for fragmentation, and 10 PASEF‐MS/MS scans were acquired per cycle. The dynamic exclusion was set to 24 s.

### LC‒MS/MS for Lactylomic Analysis

The trypsin‐digested peptides were suspended in solvent A and directly injected into a self‐made reverse‐phase analytical column (25 cm long, 100 µm i.d.). Peptide separation was carried out on a NanoElute UHPLC system (Bruker Daltonics) at a constant flow rate of 450 nL min^−1^, using the following gradient: 7–24% solvent B over 0–40 min, 24–32% solvent B over 40–52 min, 32–80% solvent B over 52–56 min, and 80% solvent B from 56–60 min.

The peptides underwent ionization via a capillary source, followed by analysis with a timsTOF Pro (Bruker Daltonics) mass spectrometer. The applied electrospray voltage was 1.60 kV. Detection of precursors and fragments was performed by a TOF detector, with MS/MS scans spanning 100–1700 m/z. The timsTOF Pro operated in PASEF mode, selecting precursors with charge states of 0–5 for fragmentation. Ten PASEF–MS/MS scans were acquired per cycle, and dynamic exclusion was set at 24s.

### Database Search

Tandem mass spectra were searched against the human Swiss‐Prot database (20389 entries, 2023.01.03) concatenated with the reverse decoy database by MaxQuant (v 1.6.15.0).^[^ ^77^ ^]^ Trypsin/P was specified as a cleavage enzyme, allowing up to 2 missing cleavages for proteome and phosphoproteome analysis and 4 missing cleavages for lactylome analysis. The mass tolerance for precursor ions was set as 20 ppm in the first search, and 20 ppm in the main search, and the mass tolerance for fragment ions was set as 20 ppm. Carbamidomethyl on Cys was specified as a fixed modification, and acetylation at the protein N‐terminus and oxidation on Met were specified as variable modifications. For phosphoproteomic samples, phosphorylation at Ser/Thr/Tyr was set as an additional variable modification. For lactylomic samples, lactylation at Lys was set as an additional variable modification. Proteins with at least 1 unique peptide with a 1% FDR at both the peptide and protein levels were selected for further analysis.

### SCNA Calling

For each tumor sample, SCNAs were called by CNVkit.^[^ ^78^ ^]^ GISTIC2.0^[^ ^79^ ^]^ was applied to identify significantly amplified or deleted focal‐level and arm‐level events, with q < 0.05 considered significant. The following parameters were used: amplification threshold = 0.1; deletion threshold = 0.1; cap value = 1.5; broad length cutoff = 0.50; remove X‐chromosome = 0; confidence level = 0.99; join segment size = 4; armlevel peel off = 1; maximum sample segments = 4000; gene GISTIC = 1.

### Mutational and CIN Signature Analysis

The nonnegative matrix factorization algorithm of Sigminer^[^ ^80^ ^]^ was used to extract mutational signatures of tumor samples. Cosine similarities were calculated between the decomposed signatures and signatures derived from COSMIC (https://cancer.sanger.ac.uk/cosmic).^[^ ^81^ ^]^ CIN signatures were quantified via the CINSignatureQuantification package (https://github.com/markowetzlab/CINSignatureQuantification) provided by Ruben et al.^[^ ^30^ ^]^

### MS Platform Quality Control

Mass spectrometry (MS) performance was periodically validated using HeLa cell lysate tryptic digests as QC standards, measured every 1–2 days. The HeLa cells, sourced from ATCC, were confirmed free of mycoplasma and authenticated via short tandem repeat analysis. The QC samples were processed using identical protocols, conditions, software, and settings as the HeLa samples. Pairwise Pearson's correlation coefficients were computed and are displayed in Figure S1C (Supporting Information).

### Preprocessing of Proteomic, Phosphoproteomic, and Lactylomic Data

Proteome quantification was conducted via the iBAQ algorithm, and the phosphoproteome and lactylome were quantified at the site level. All quantification data were normalized to the median of each sample to correct potential sample loading differences and then multiplied by the global median of each omic dataset for ease of presentation and log2 transformation. Protein, phosphosite, and lactylomic data with more than 50% missing values in both the tumor and NAT samples were excluded from further analysis. K‐nearest neighbor (KNN) imputation was applied to impute the missing values via the R package DreamAI.^[^ ^82^ ^]^

### Differential Expression Analysis and Overrepresentation Analysis

Differential expression analysis was conducted via the Wilcoxon rank‐sum test. Statistical significance was considered when the *p* value was < 0.05. Proteins and modifications significantly upregulated and downregulated in tumor tissues were then subjected to functional enrichment analyses via the ConsensusPathDB (http://cpdb.molgen.mpg.de/).^[^ ^83^ ^]^

### Correlation of Lactylation and Protein Abundance

To compare lactylation and protein abundance, the MPRs were calculated on the basis of the calculation method used for the PTRs.^[^ ^32^, ^33^ ^]^ The MPR values followed a Gaussian distribution, similar to PTRs in a previous report.^[^ ^33^ ^]^ High‐MPR or low‐MPR PTM sites were defined as MPR > median + SD or MPR < median – SD.

### Histone Lactylation Regulation Analysis

To examine the links between acyltransferase/deacylase and histone lactylation levels, a Lasso regression model was applied. In this model, acyltransferases/deacylases and histone protein expression were set as independent variables, while a histone lactylation site served as the dependent variable, following a prior study.^[^ ^84^ ^]^ A total of 300 bootstraps were executed using 80% training data and 20% testing data, presenting the average coefficients from the 300 iterations. Proteins with average coefficients below 0.1 were excluded.

Spearman's correlation analysis was also carried out between histone lactylation sites and hallmark GSVA scores to uncover the potential epigenetic regulation of histone lactylation.

### GSEA

GSEA software (v4.3.2) (http://software.broadinstitute.org/gsea/index.jsp) was used to perform GSEA^[^ ^85^ ^]^ on the basis of the KEGG, WikiPathways, and HALLMARK gene sets downloaded from MSigDB v7.1 (http://software. broadinstitute.org/gsea/msigdb/index.jsp). A *p* value < 0.05 was used as a cutoff. The normalized enrichment score was used to reflect the degree of pathway overrepresentation.

### Kinase Activity Analysis

The inference of kinase activity was based on the kinase‒substrate relationships obtained from PhosphoSitePlus and NetworKIN.^[^ ^86^ ^]^ The predicted substrates with a NetworKIN score > 5 were retained. Kinase activity in each sample was calculated via the ssGSEA method in the GSVA package. KSEA was also performed using the PhosphoSitePlus and NetworKIN databases via the KSEA app (https://casecpb.shinyapps.io/ksea/).^[^ ^87^ ^]^ Kinases with an FDR < 0.05 were considered significant.

### Transcription Factor Activity Analysis

Transcription factor activity inference was based on the TF‐target gene relationships obtained from DoRothEA.^[^ ^88^ ^]^ The TF‐target gene relationships with A, B, and C confidence values were retained. The TF activity in each sample was calculated via VIPER.^[^ ^89^ ^]^ In addition, the TF‐target relationships were also used as gene sets for GSEA. The target genes of *TP53* were also obtained from DoRothEA.

### Molecular Subtyping of HER2‐Low Breast Tumors

For proteomic subtyping, consensus clustering was executed using the R package CancerSubtypes (v.1.22.1),^[^ ^90^ ^]^ employing Spearman's correlation coefficient as the distance metric. The process involved 1000 bootstraps, an item subsampling proportion of 0.8, and a feature subsampling proportion of 1. Proteins with the highest 50% median absolute deviation (MAD) in HER2‐low tumor samples were selected for hierarchical clustering into up to six groups. Consensus matrices for k = 2 to 6 are displayed in Figure S5A (Supporting Information), with k = 3 showing distinct cluster separation. Cumulative distribution functions and metrics like relative changes in area under the curve and average silhouette distance aided in selecting the optimal k value (Figure S5B,C, Supporting Information).

In lactylomic subtyping, k‐means clustering, based on Euclidean distance, was applied to lactylation sites with the highest 50% MAD in tumor samples. Other parameters mirrored those in proteomic subtyping. Relevant consensus matrices, cumulative distributions, area changes, and average silhouette distances are presented in Figure S8A–C (Supporting Information).

### SVM Classifier for the HER2‐Low Proteomic Subtype

Before model construction, the proteomic data were scale‐normalized. GSEA was conducted to identify signature proteins of each proteomic subtype, and the top 20 proteins with the highest and lowest scores in each subtype were selected. A support vector machine recursive feature elimination (SVM‐RFE) model was trained on 70% of the HER2‐low tumor samples. The remaining 30% of the samples were used as the test set. Fivefold cross validation was used for hyperparameter optimization.

CPTAC breast proteomic data were downloaded from LinkedOmics^[^ ^91^ ^]^ (https://linkedomics.org/data_download/CPTAC‐BRCA/). The proteomic data from the CPTAC cohort were further preprocessed via median centering, KNN imputation, and scale normalization. Only HER2‐negative tumors were used for HER2‐low proteomic subtype classification.

### Tamoxifen Treatment Response Prediction

The proteome data and tamoxifen treatment response data were derived from Marchi et al.^[^ ^41^ ^]^ After KNN imputation, the Wilcoxon rank sum test was conducted to calculate the significance of the difference. Only proteins identified in the study were retained. The dataset was split into a training set (80%) and a test set (20%). The top 100 most significantly differentially expressed proteins were used for further model construction. A random forest recursive feature elimination (RF‐RFE) approach was used for feature selection. The hyperparameters were optimized via fivefold cross‐validation in the training set.

### Tumor Microenvironment Analysis

The immune score, stromal score, and tumor purity were inferred via the R package ESTIMATE (v1.0.11).^[^ ^92^ ^]^ To evaluate the tumor immune microenvironment of breast tumors, the raw enrichment scores of 64 different cell types were computed via xCell^[^ ^44^ ^]^ on the basis of the tumor proteomic profiles. Cell types (such as hepatocytes, neurons, and astrocytes) that do not exist in breast or breast tumor tissues were excluded.

### IHC Analysis

Paraffin‐embedded tissue sections were deparaffinized at 60 °C for 20 min, cleared in xylene, and subjected to an alcohol gradient. IHC required a heat treatment via saline‒sodium citrate buffer at 95–100 °C. Following cooling, the slides were blocked at room temperature with blocking solution (2% goat serum, 2% BSA, and 0.05% Tween in PBS) for 2 h and incubated with a primary antibody diluted in blocking solution overnight at 4 °C. Endogenous peroxidase activity was quenched with 0.3% H_2_O_2_. The slides were then incubated with a horseradish peroxidase (HRP)‐conjugated secondary antibody (Abbkine, A21020/A21030) at room temperature, followed by development with a 3,3′‐diaminobenzidine substrate (ZSGB‐BIO, ZLI‐9018). Counterstaining was performed using hematoxylin, and dehydration was carried out using an alcohol gradient. The densities were determined by counting positive cells in a ×10 high‐power field of view (≈2 mm^2^). The following antibodies were used: COL16A1 (Abcam, ab231044); SBSPON (Atlas Antibodies, HPA029595); VAV3 (Novus, NB300‐817); CREBBP (Atlas Antibodies, HPA055861); ELP1 (Atlas Antibodies, HPA050686); RFC1(Abcam, ab180613); HEATR5B (Atlas Antibodies, HPA042025); XPO5 (Atlas Antibodies, HPA023959); TFDP1 (Abcam, ab186831); and TOM1L2 (Atlas Antibodies, HPA022541). To ensure reproducibility and adjudication accuracy, every slide was independently reviewed by two board‐certified breast pathologists who were blinded to clinical data. Discordant results (<5% of cases) were resolved by consensus after multi‐head microscopy evaluation with a third senior pathologist.^[^ ^75^ ^]^

### Patient‐Derived Organoid Establishment

To construct the patient‐derived organoid (PDO) model, fresh tumor tissues were collected in ice‐cold Hanks' balanced salt solution (Gibco, Catalog #14 175 095) supplemented with antibiotics, minced, and digested with collagenase type II (Gibco, Catalog #17 101 015) at 2 mg mL^−1^ for 1 h at 37 °C. After centrifugation at 300 × g for 5 min, the supernatant was discarded, and the pellet was resuspended in 1640 (Gibco, Catalog # 11 875 093) medium. The cell suspension was filtered through a 100 µm cell strainer (Bioshark, Catalog # BS‐100‐CS) to remove debris, mixed with cold Matrigel (Corning, Catalog #356 231), and plated in 24‐well plates. The Matrigel drops were solidified at 37 °C for 15‒30 min and then overlaid with OrganoPro medium (K2 ONCOLOGY, Catalog # K2O‐M‐BR). Organoids were harvested and dissociated into single cells, and approximately 2000 cells were plated in each well of a low‐adhesion 96‐well plate (Corning, Catalog #3474) coated with Matrigel. For drug administration, therapeutic agents were dissolved to prepare stock solutions, which were then diluted in the organoid culture medium. After solidification, organoid culture medium containing various drug concentrations was added, with control wells receiving equivalent concentrations, and the plates were incubated at 37 °C for 48 h. Cell viability was measured via an Enhanced Cell Counting Kit‐8 (Bioshark, Catalog # BL1055C). Equal volumes of Enhanced Cell Counting Kit‐8 reagent were added to each well after incubation with medium at a ratio of 1:10. The mixture was incubated at room temperature for 1 h, and the optical density was measured via an Enspire 2300 microplate reader.

### Statistical Analysis

Data are presented as mean ± SEM from six or nine independent experiments. Multiple comparisons were performed with one‐way ANOVA followed by Bonferroni (equal variances) or Dunnett's T3 (unequal variances) post‐hoc tests in SPSS 29.0 (SPSS Inc., Chicago, IL, USA). A *p*‐value < 0.05 was taken as statistically significant.

## Conflict of Interest

The authors declare no conflict of interest.

## Supporting information



Supporting Information

## Data Availability

The data that support the findings of this study are available from the corresponding author upon reasonable request.
