# Supplementary material for: Proteogenomic Characterization Reveals Subtype‐Specific Therapeutic Potential for HER2‐Low Breast Cancer
Source: Adv Sci (Weinh). 2025 Dec 27;13(12):e13086. doi: 10.1002/advs.202513086 (PMC12948274; doi:10.1002/advs.202513086)
Supplement: Supplementary file 1 — Supporting Information [file ADVS-13-e13086-s001.docx]

**Proteogenomic characterization reveals subtype-specific therapeutic potential for HER2-low breast cancer**

Shouping Xu^1,2,8^, Keda Yu^3,8^, Lei Liu^1,8^, Qin Wang^2,4,8^, Xiaohui Wu^5,8^, Yihai Chen^1^, Guozheng Li^1^, Xin Zhang^1^, Bo Wei^1^, Zitong Fu^1^, Abiyasi Nanding^6^, Zuxianglan Zhao^5^, Lingbing Yang^1^, Xingda Zhang^1,2,4^, Jianyu Wang^1,2,4^, Wantong Sun^1^, Yi Hao^1^, Zhongyi Cheng^5*^, Xiaojiang Cui^7*^, Hao Wu^2,4*^, Da Pang^1,4,9*^

^1^ Department of Breast Surgery, Harbin Medical University Cancer Hospital, Harbin 150081, China.

^2^ Key Laboratory of Tumor Biotherapy of Heilongjiang Province, Harbin Medical University Cancer Hospital, Harbin, 150081, China.

^3^ Department of Breast Surgery, Shanghai Cancer Center and Cancer Institute, Shanghai Medical College, Fudan University, Shanghai 200032, China.

^4^ Heilongjiang Clinical Research Center for Breast Cancer, Harbin Medical University Cancer Hospital, Harbin, 150081, China.

^5^ Jingjie PTM Biolab (Hangzhou) Co. Ltd., Hangzhou, 310018, China

^6^ Department of Pathology, Harbin Medical University Cancer Hospital, Harbin 150081, China.

^7^ Department of Surgery, Samuel Oschin Cancer Institute, Cedars-Sinai Medical Center, Los Angeles, CA 90048, USA.

^8^ These authors contributed equally.

^9^ Lead contact.

*Correspondence:

[pangda@ems.hrbmu.edu.cn](mailto:pangda@ems.hrbmu.edu.cn) (D.P.) (Lead contact);

[3345@hrbmu.edu.cn](mailto:3345@hrbmu.edu.cn) (H.W.);

[xiaojiang.cui@cshs.org](mailto:xiaojiang.cui@cshs.org) (X.C.).

[zhongyi_cheng@ptm-biolab.com](mailto:zhongyi_cheng@ptm-biolab.com) (Z.C.)


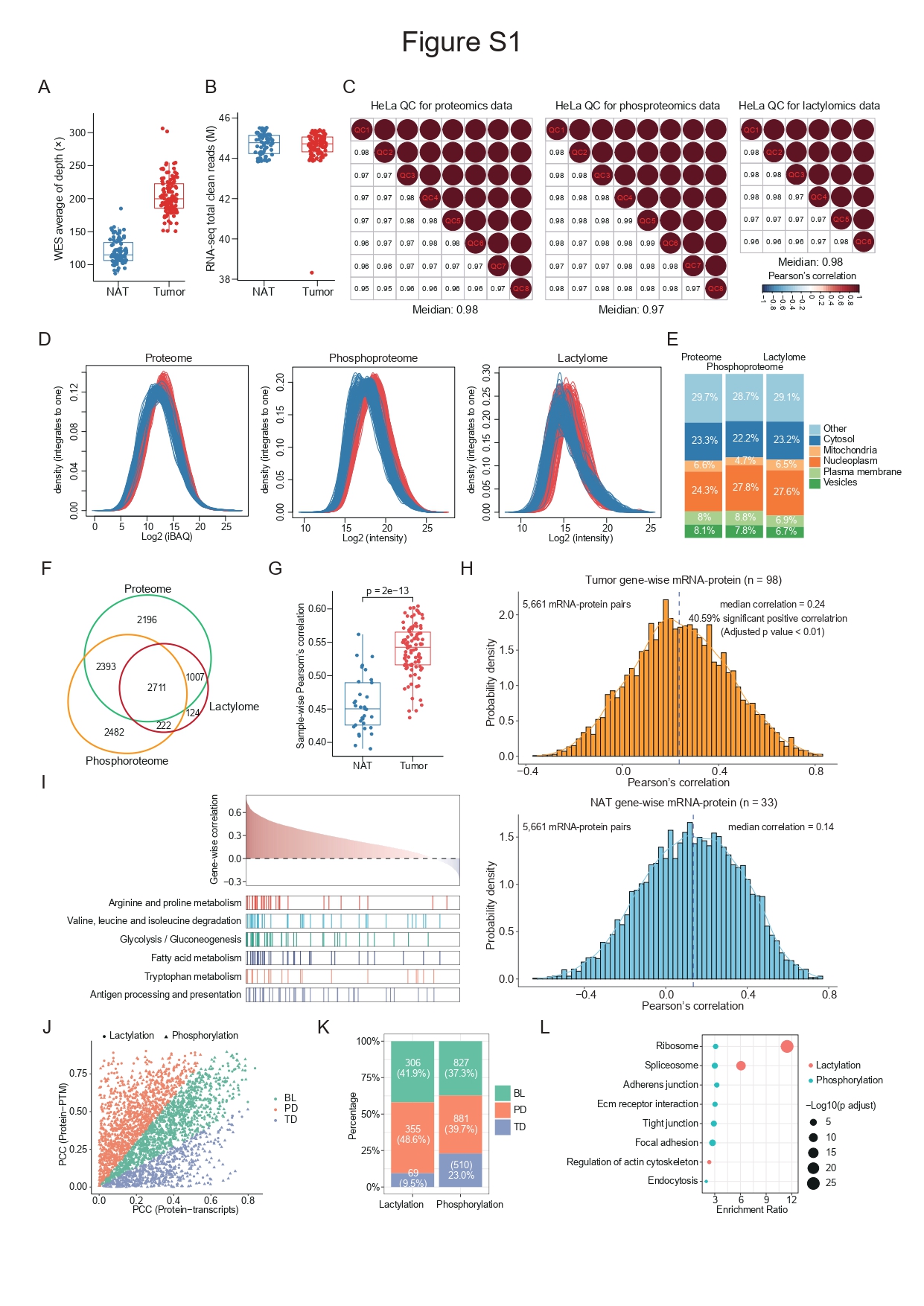


**Fig. S1** **Data quality assessments of multi-omics, related to Fig. 1**

1. Mean coverage of WES in breast cancer tumor and normal adjacent tissues.
2. QC-passed reads in RNA-seq of breast cancer tumor and normal adjacent tissues.
3. Longitudinal quality control of the mass spectrometry platform for proteome, phosphoproteome, and lactylome analysis.
4. Density plots showing the quantification of the proteome, phosphoproteome, and lactylome.
5. Bar plots showing the subcellular locations of the identified proteins at the proteome, phosphoproteome, and lactylome levels.
6. Venn diagram showing the overlapping proteins identified at the proteome, phosphoproteome, and lactylome levels.
7. Boxplots showing the samplewise mRNA‒protein Pearson’s correlations. Boxplots show the median (central line), the 25–75% IQR (box limits), and the ±1.5×IQR (whiskers).
8. Gene-wide mRNA‒protein correlations in tumor and NAT samples.
9. Functional enrichment via gene‒wide mRNA‒protein correlation in tumors.
10. The definition of contribution ratio of PTM and transcript (PTC).
11. The proportions of proteins from the TD, BL and PD categories at phosphorylation and lactylation levels, respectively.

KEGG pathway enrichment results of PD proteins at phosphorylation and lactylation levels.


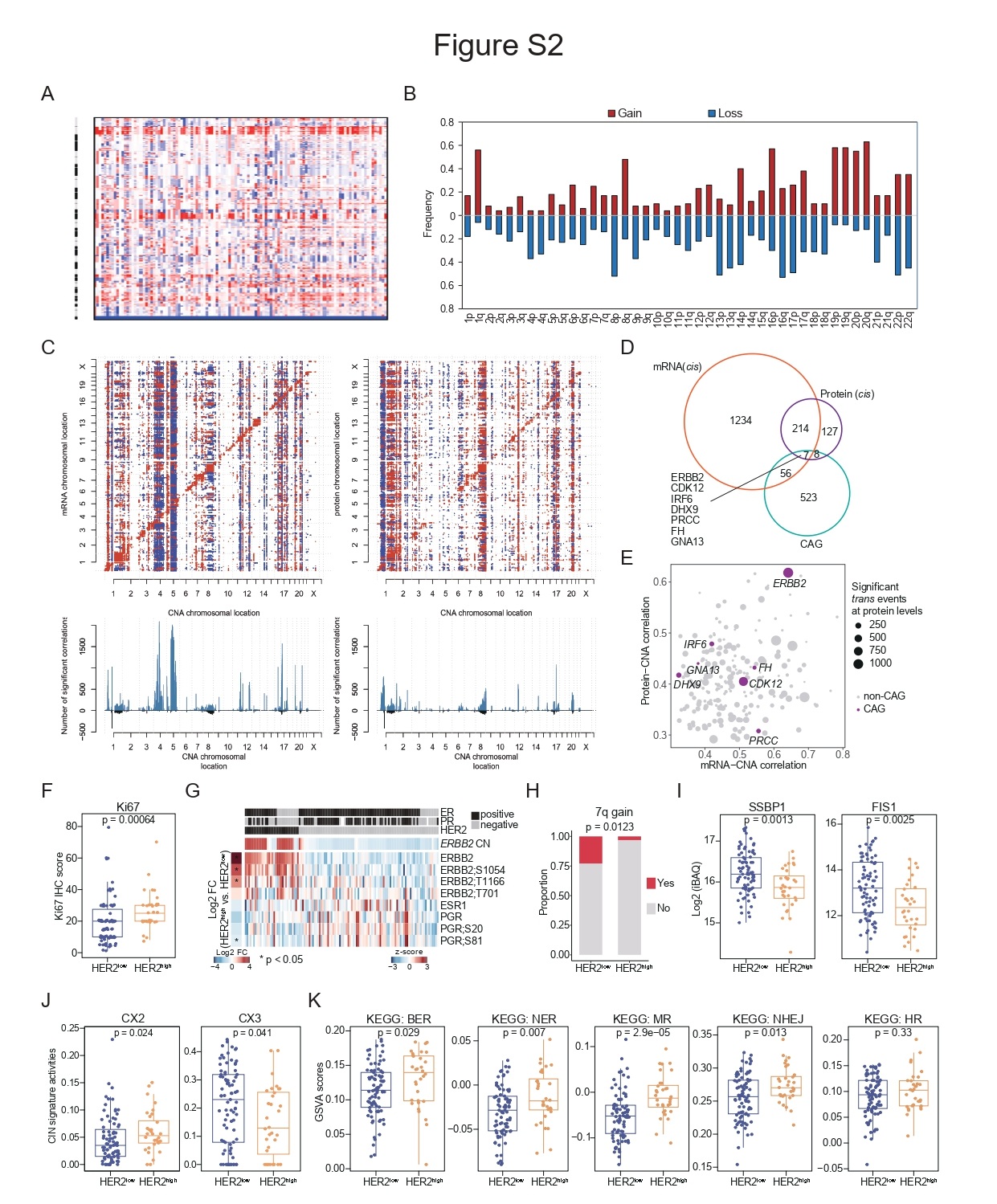


**Fig. S2 Proteogenomic features of HER2-low breast cancer, related to Fig. 2**

1. SCNA profiles of the breast cancers.
2. Arm-level SCNA events. Red denotes gain, and blue denotes loss.
3. Upper: Heatmaps showing correlations between copy number alterations and transcriptomic (left) or proteomic (right) data. Red and blue events represent significant (Spearman’s correlation, FDR < 0.05) positive and negative correlations, respectively. Lower: number of mRNAs or proteins that were significantly associated with a specific CNA. The gray bars indicate correlations specific to mRNAs or proteins, and the black bars indicate correlations with both mRNAs and proteins.
4. Venn diagram showing the overlap of cancer-associated genes (CAGs) with significant cis effects (FDR < 0.05) observed at the RNA and protein levels.
5. Scatter plot showing the trans-regulation of CNA events with significant cis effects at both the RNA and protein levels. CAGs are indicated in purple.
6. Comparison of Ki67 IHC scores between HER2-high and HER2-low patients (Wilcoxon rank-sum test).
7. Heatmap showing the comparisons of the IHC score, copy number alteration, protein level, and phosphosites of ERBB2, ESR1, and PGR between HER2-high and HER2-low tumors.
8. Comparison of 7q gain events between HER2-high and HER2-low tumors (Fisher’s exact test).
9. Comparisons of SSBP1 and FIS1 protein abundances between HER2-high and HER2-low tumors (Wilcoxon rank-sum test).
10. Comparisons of CX2 and CX3 signature activities in HER2-high and HER2-low tumors.
11. Comparisons of DNA repair pathways, including BER (base excision repair), NER (nucleotide excision repair), MR (mismatch repair), NHEJ (nonhomologous end-joining), and HR (homologous recombination), between HER2-high and HER2-low tumors (Wilcoxon rank-sum test).


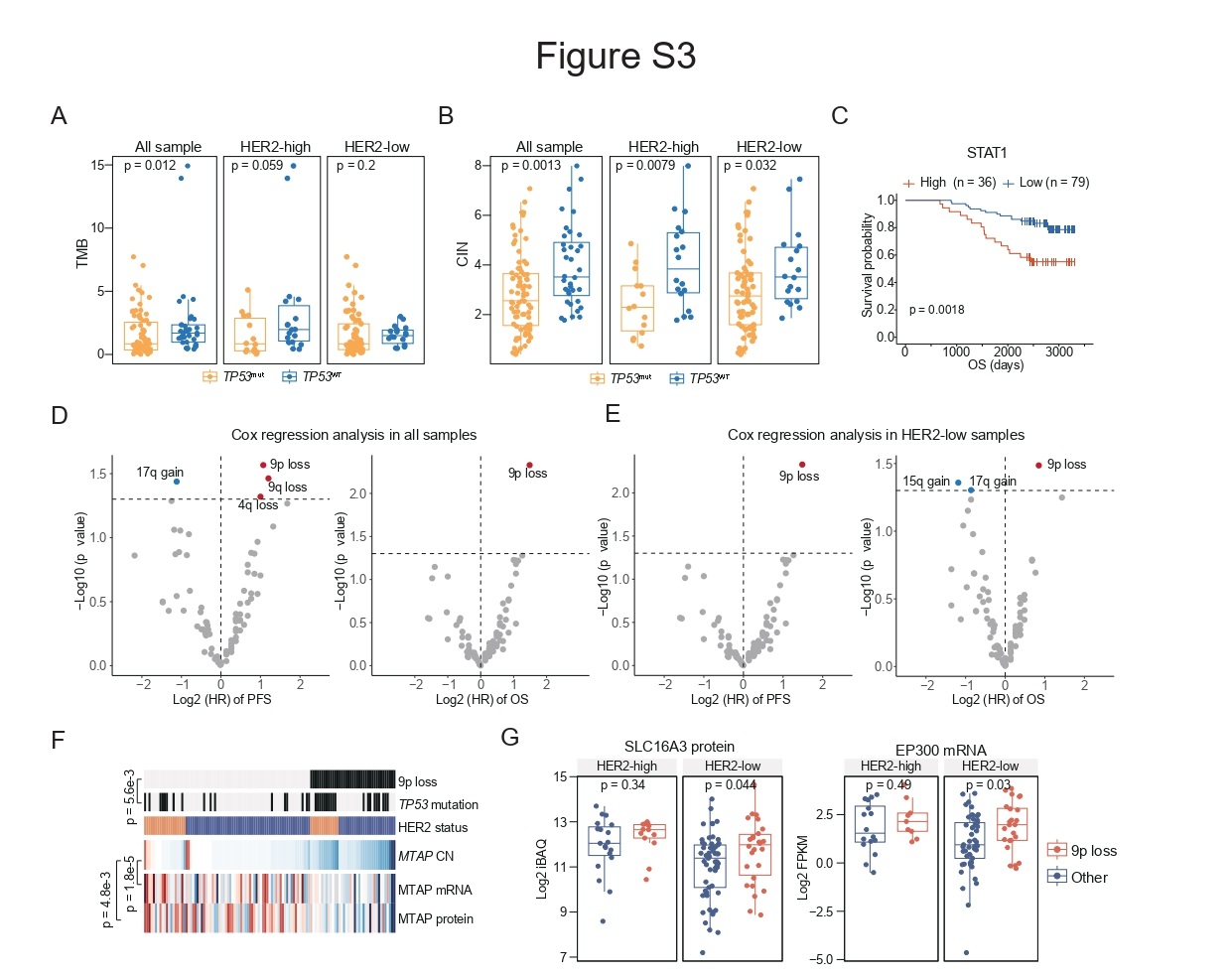


**Fig. S3** **Prognosis associated genomic alterations in HER2-low breast cancers, related to Fig. 3**

1. Comparisons of TMB between tumors with and without TP53 mutation in HER2-high and HER2-low tumors (Wilcoxon rank-sum test).
2. Comparisons of CIN between tumors with and without TP53 mutation in HER2-high and HER2-low tumors (Wilcoxon rank-sum test).
3. Kaplan–Meier curves of OS for patients with different STAT1 protein levels (two-sided log-rank test).
4. Cox regression analysis of arm-level CNA events based on PFS and OS in all samples in this study.
5. Cox regression analysis of arm-level CNA events based on PFS and OS in HER2-low samples.
6. Cis-regulation of MTAP by 9p loss in this study.

Comparisons of SLC16A3 and EP300 expression levels between HER2-high and HER2-low tumors with and without 9p loss.


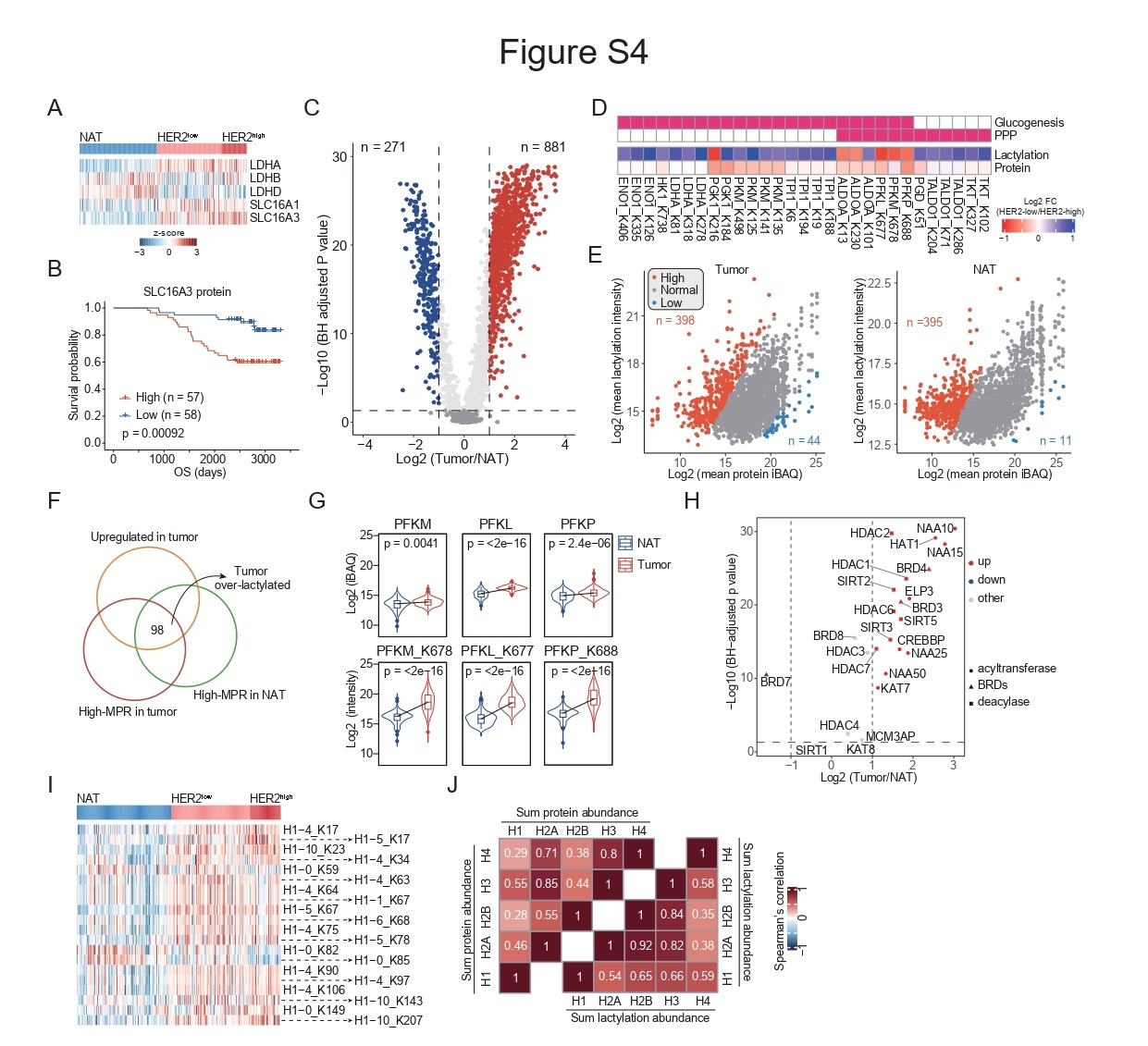


**Fig. S4** **Lactylation alterations of HER2-low breast cancers, related to Fig. 4**

1. Heatmap showing the protein abundances of LDHA, LDHB, LDHD, SLC16A1, and SLC16A3 in tumors and NATs.
2. Kaplan–Meier curves of OS for patients with different SLC16A3 protein levels (two-sided log-rank test).
3. Volcano plot showing the differentially lactylated sites between normal adjacent and tumor tissues.
4. Heatmap showing the differential glycolytic enzyme protein and lactylation levels between HER2-low and HER2-high tumors.
5. Scatter plot of lactylation versus protein abundance in tumor and NAT samples. Red and blue mark the lactylation sites with high and low MPRs, respectively, as defined.
6. Venn diagram showing the overlap of tumor-upregulated lactylation sites, high-MPR sites in tumors, and high-MPR sites in NATs.
7. Protein and lactylation levels of PFKM, PFKL, and PFKP in tumors and NATs.
8. Differential protein expression of acyltransferases, deacylases, and bromodomain-containing proteins between tumors and NATs.
9. Heatmap showing the linker histone lactylation between tumors and NATs.

Correlations of histone protein abundance and lactylation levels.


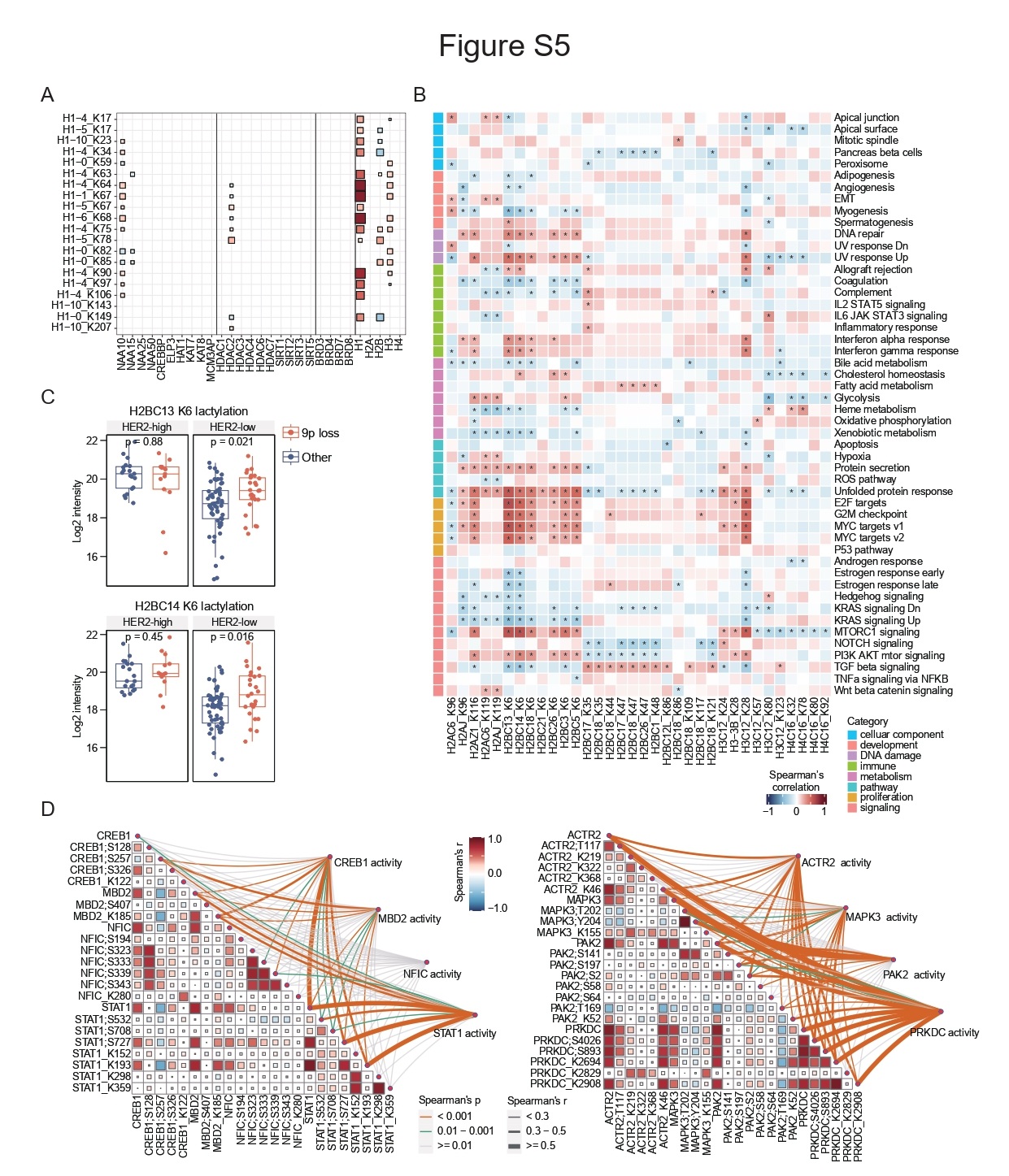


**Fig. S5** **Histone, TF, and kinase lactylations, related to Fig. 4**

1. Significant associations between linker histone lactylation sites and acyltransferases, deacylases, and bromodomain-containing proteins.
2. Heatmap showing the correlations between histone lactylation and hallmark pathways. The correlations with p < 0.05 are indicated by asterisks.
3. Comparisons of H2BC13 and H2BC14 K6 lactylation levels between tumors with and without 9p loss in HER2-high and HER2-low tumors.

Correlation analysis of TF and kinase activities among lactylation levels and proteomic and phosphoproteomic levels.


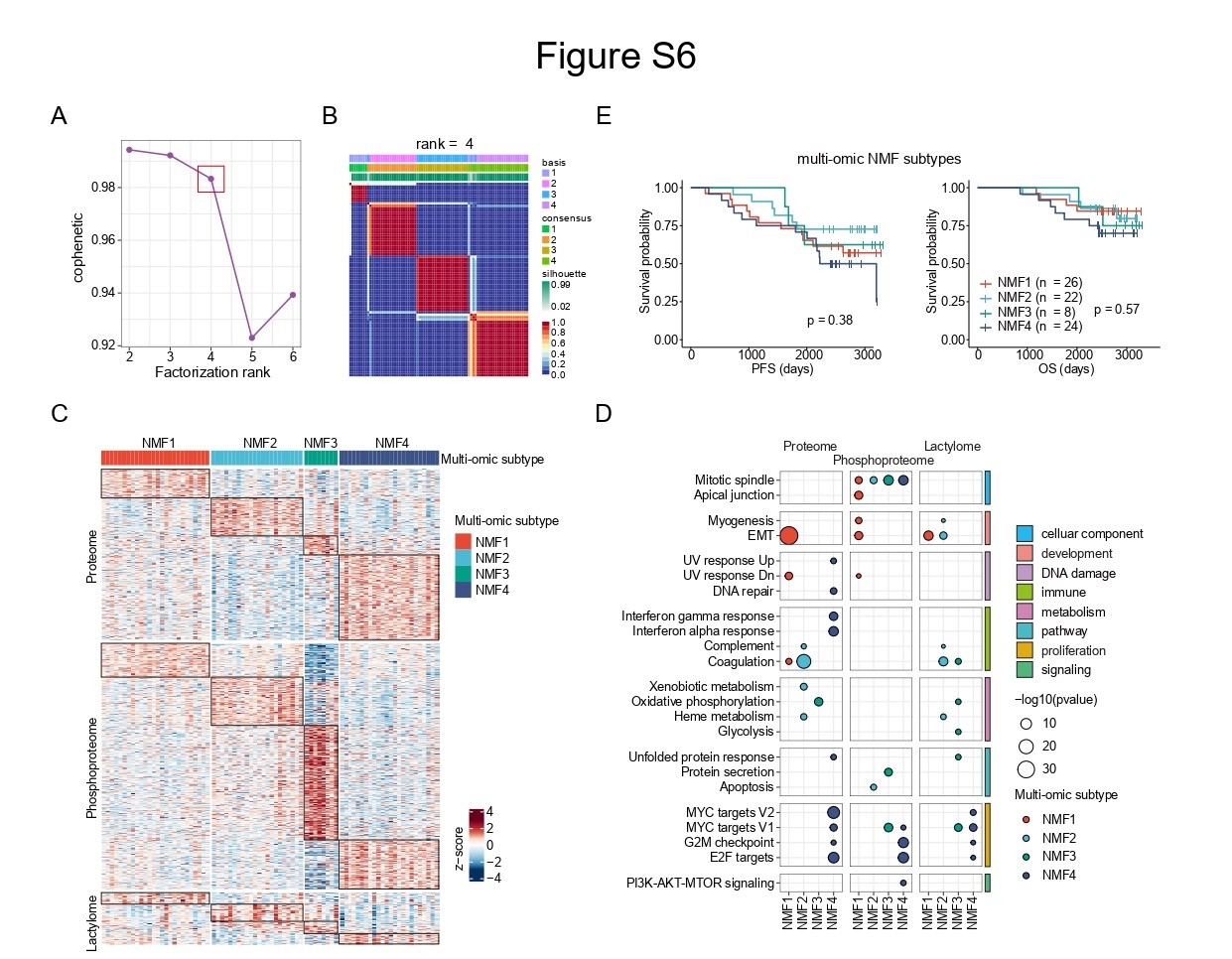


**Fig. S6** **Multi-omic subtyping of HER2-low breast cancers**

1. Cophenetic correlation for NMF clustering as a function of the number of clusters K.
2. Consensus heatmap of NMF clustering at rank = 4.
3. Relative abundances of upregulated proteins, phosphosites, and lactylation sites in the NMF multiomic subtypes and associations of multiomic subtypes with proteomic and lactylomic subtypes.
4. The upregulated hallmarks pathways of multiomic subtypes at proteome, phosphoproteome, and lactylome levels.
5. Kaplan–Meier curves of PFS (left) and OS (right) for the NMF multi-omic subtypes (two-sided log-rank test).


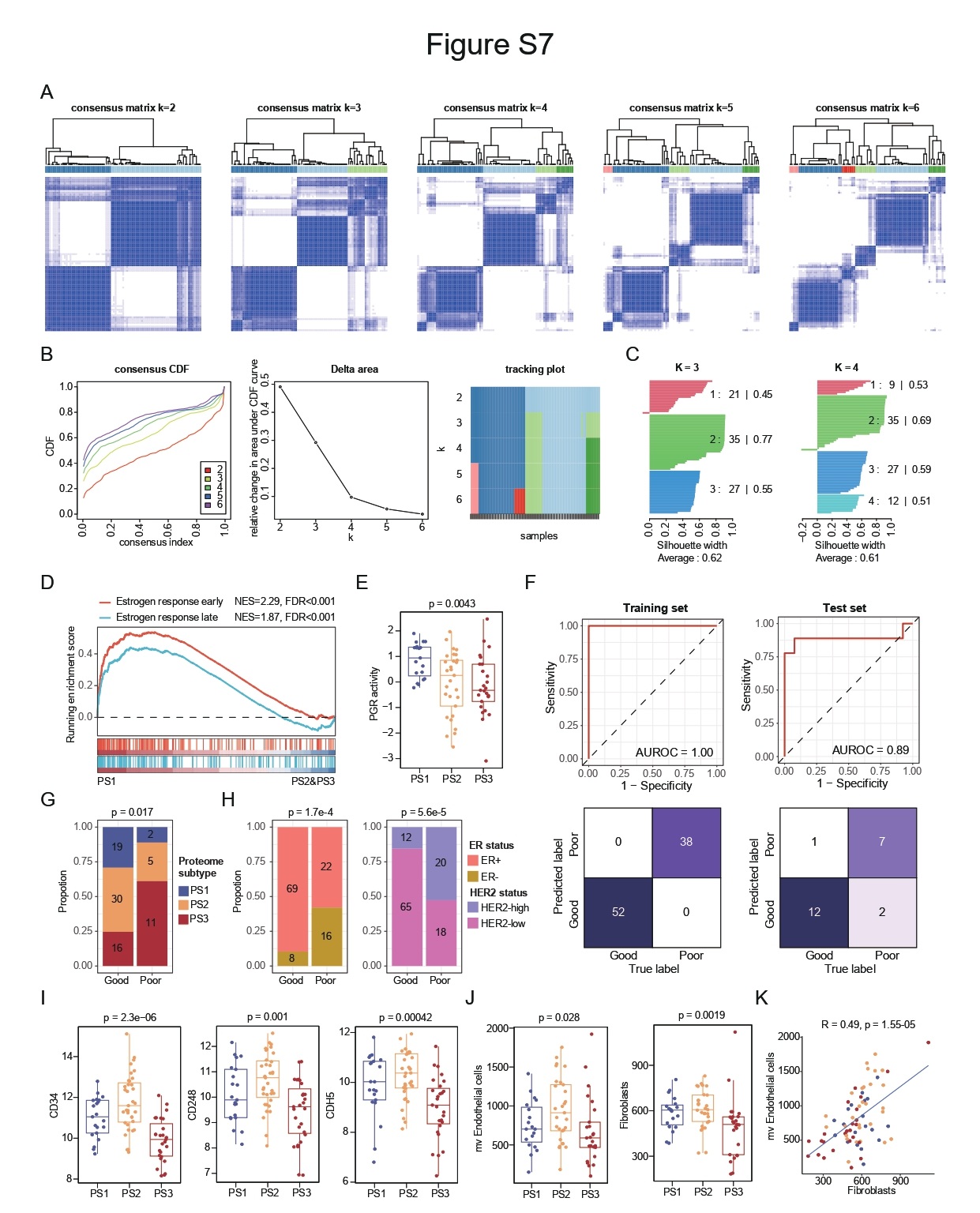


**Fig. S7** **Consensus clustering of HER2-low breast cancers, related to Fig. 5**

1. Consensus matrices of 83 HER2-low breast cancer tumor samples from k = 2 to k = 6 via proteomic data.
2. Cumulative distribution function plot, delta plot, and tracking plot corresponding to consensus matrices from k = 2 to k = 6.
3. Silhouette plots (k = 3, 4).
4. GSEA plot showing a greater estrogen response in PS1 tumors than in PS2 and PS3 tumors at the transcriptomic level.
5. Boxplots showing the comparisons of PGR activity, inferred by VIPER via transcriptome data, among the three proteomic subtypes (Kruskal‒Wallis test).
6. The performance of the tamoxifen response classifier in the training and test sets. The ROC curves and confusion matrix are shown.
7. Comparison of the predicted tamoxifen response among the three proteomic subtypes (Fisher’s exact test).
8. Comparison of the predicted tamoxifen response between patients with distinct ER or HER2 statuses (Fisher’s exact test).
9. Boxplots showing the comparisons of protein abundances of tumor vascular markers (CD34, CD248, and CDH5) among the three proteomic subtypes (Kruskal‒Wallis test).
10. Boxplots showing the comparisons of mv endothelial cell and fibroblast xCell scores among the three proteomic subtypes (Kruskal‒Wallis test).
11. Scatter plot showing the correlation between MV endothelial cell and fibroblast xCell scores (Spearman’s correlation).


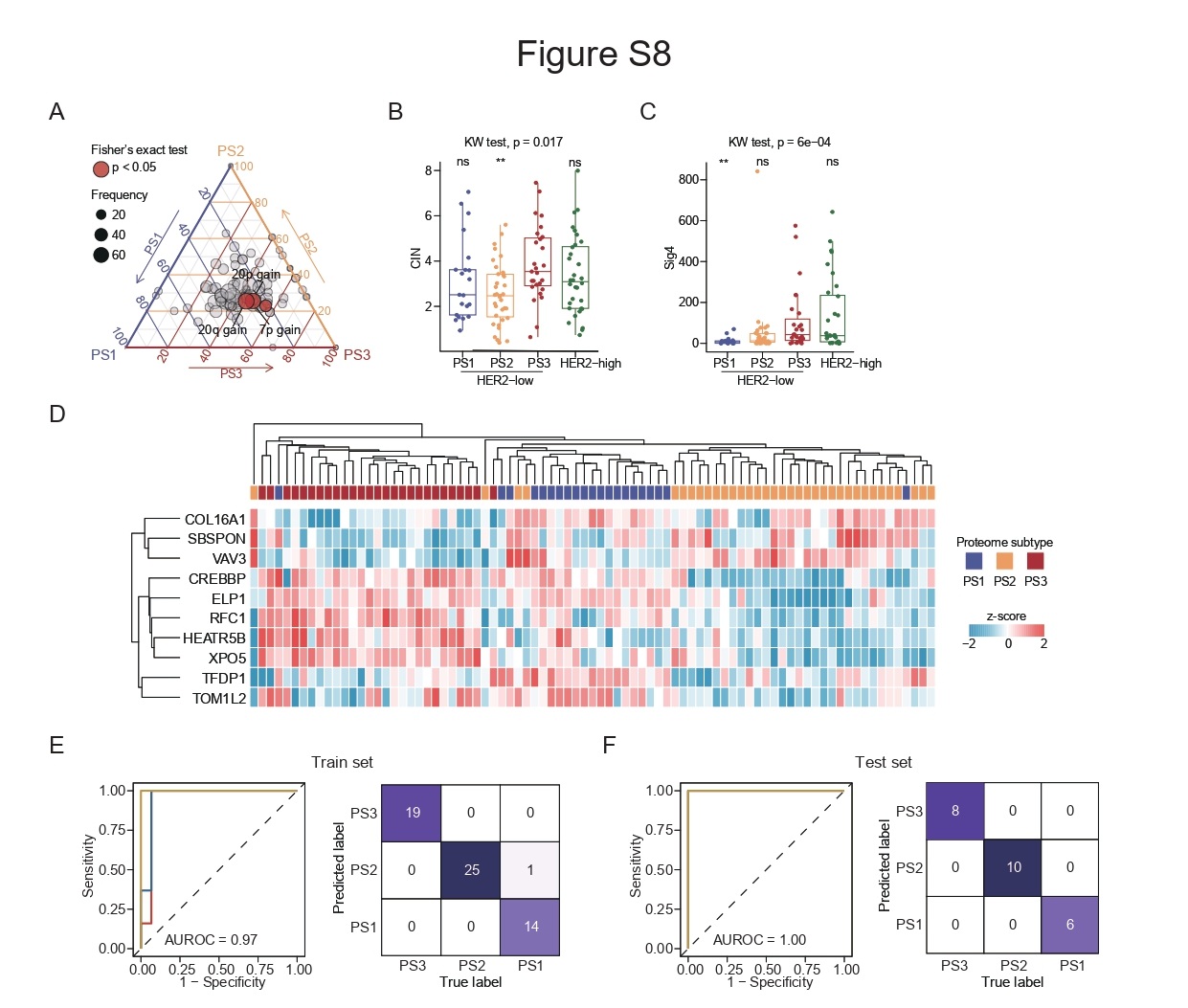


**Fig. S8** **Predictor of the HER2-low breast cancer subtypes, related to Fig. 5**

1. Ternary plot showing the distribution of arm-level events in the three proteomic subtypes. CNA events with significant differences among the three proteomic subtypes are indicated (Fisher’s exact test).
2. Comparisons of CIN among three HER2-low proteomic subtypes and HER2-high tumors. The Kruskal‒Wallis test and Wilcoxon rank‒sum test were performed. * p < 0.05, ** p < 0.01, ns p ≥ 0.05.
3. Comparisons of Sig4 activities among three HER2-low proteomic subtypes and HER2-high tumors. The Kruskal‒Wallis test and Wilcoxon rank‒sum test were performed. * p < 0.05, ** p < 0.01, ns p ≥ 0.05.
4. Heatmap of 10 protein signatures that discriminate among the three proteomic subtypes.
5. The performance of the proteomic subtype classifier in the training set. The ROC curve and confusion matrix are shown.

The performance of the proteomic subtype classifier in the test set. The ROC curve and confusion matrix are shown.


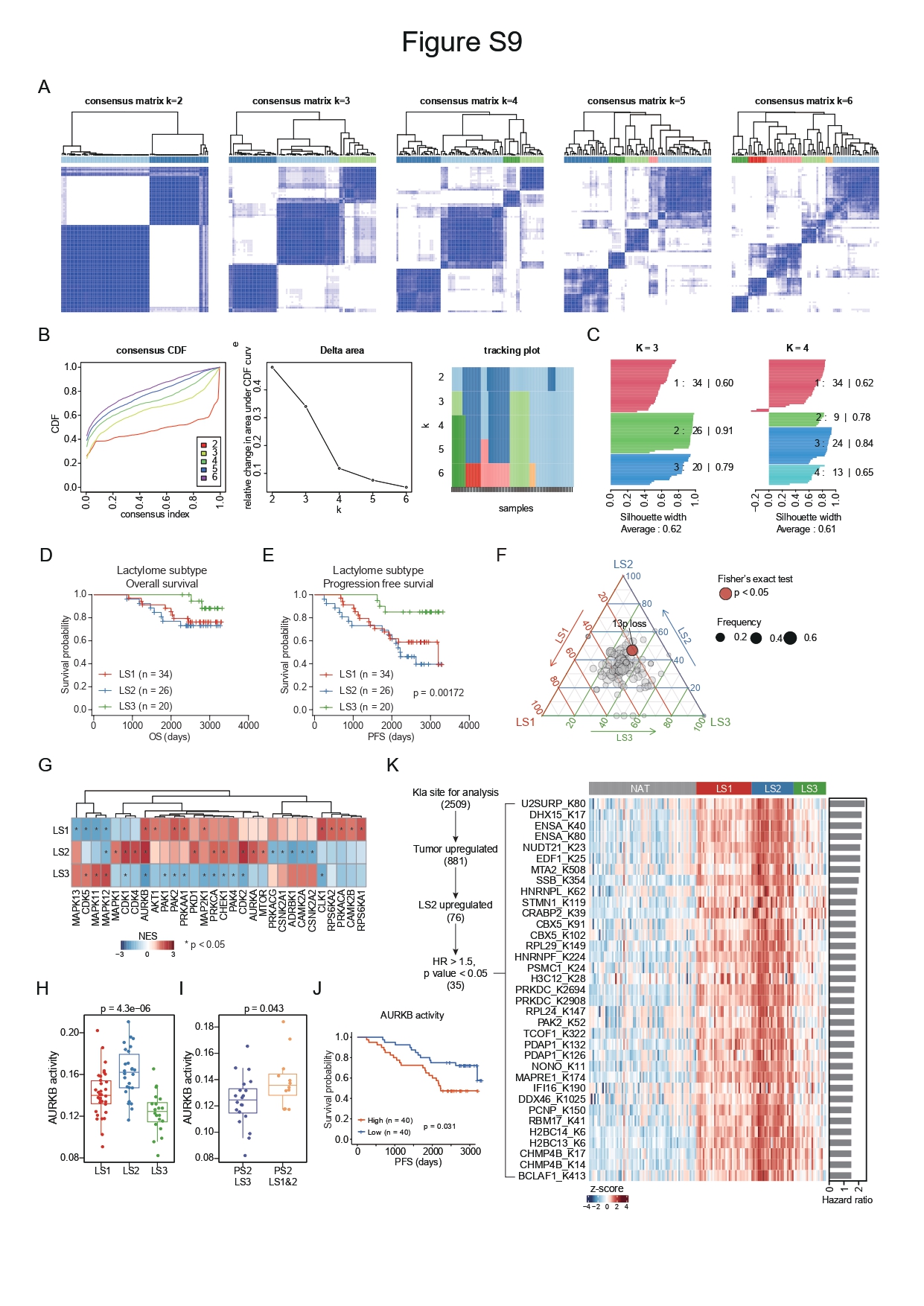


**Fig. S9** **Deciphering the PTM heterogeneity in HER2-low breast cancer, related to Fig. 6**

1. Consensus matrices of the 80 HER2-low breast cancer samples from k = 2 to k = 6 via lactylomic data.
2. Cumulative distribution function plot, delta plot, and tracking plot corresponding to consensus matrices from k = 2 to k = 6.
3. Silhouette plots (k = 3, 4).
4. Kaplan–Meier curves of OS for the three lactylome subtypes (two-sided log-rank test).
5. Kaplan–Meier curves of PFS for the three lactylome subtypes (two-sided log-rank test).
6. Ternary plot showing the distribution of arm-level events in the three lactylome subtypes. CNA events with significant differences among the three lactylome subtypes are indicated (Fisher’s exact test).
7. Kinase-substrate enrichment analysis among the three lactylation subtypes.
8. Comparisons of AURKB activities among three HER2-low lactylation subtypes (Kruskal‒Wallis test).
9. Comparisons of AURKB activities between PS2-LS1&2 and PS2-LS3 tumors (Wilcoxon rank-sum test).
10. Kaplan–Meier curves of PFS for patients with different AURKB activities.
11. Left: Strategy for prioritizing targets in LS2-upregulated lactylation. Right: Heatmap of relative lactylation abundances among lactylation subtype tumors and NATs.
